# Novel Strategies to Combat CMV-Related Cardiovascular Disease

**DOI:** 10.20411/pai.v5i1.382

**Published:** 2020-09-20

**Authors:** Elena Vasilieva, Sara Gianella, Michael L. Freeman

**Affiliations:** 1 Laboratory of Atherothrombosis, Moscow State University of Medicine and Dentistry, Moscow 127473, Russia; 2 Division of Infectious Diseases and Global Public Health, Department of Medicine, University of California, San Diego, La Jolla, CA 92093, USA; 3 Division of Infectious Diseases and HIV Medicine; Department of Medicine; Case Western Reserve University, Cleveland, Ohio, United States

**Keywords:** Cytomegalovirus (CMV), Cardiovascular Disease (CVD), Human Immunodeficiency Virus (HIV), Atherosclerosis, Inflammation, Antivirals, Immunotherapy, Vaccines

## Abstract

Cytomegalovirus (CMV), a ubiquitous human pathogen that is never cleared from the host, has long been thought to be relatively innocuous in immunocompetent adults, but causes severe complications including blindness, end-organ disease, and death in newborns and in immuno-compromised individuals, such as organ transplant recipients and those suffering from AIDS. Yet even in persons with intact immunity, CMV infection is associated with profound stimulation of immune and inflammatory pathways. Carriers of CMV infection also have an elevated risk of developing cardiovascular complications. In this review, we define the proposed mechanisms of how CMV contributes to cardiovascular disease (CVD), describe current approaches to target CMV, and discuss how these strategies may or may not alleviate cardiovascular complications in those with CMV infection. In addition, we discuss the special situation of CMV coinfection in people with HIV infection receiving antiretroviral therapy, and describe how these 2 viral infections may interact to potentiate CVD in this especially vulnerable population.

## INTRODUCTION

Human cytomegalovirus (CMV) is a beta-herpesvirus that is common worldwide, infecting about 50% of the general population in the United States and in Europe, and is particularly prevalent among those who have low socio-economic status or receptive sexual intercourse (women and men who have sex with men) [1, 2]. Many adults are initially infected with CMV during childhood or early adulthood, and the incidence continues to rise throughout adulthood, by approximately 1% annually [3]. In some resource-poor areas, such as sub-Saharan Africa and parts of Asia, CMV infection rates approach 100% [4-6]. Primary CMV infection elicits robust innate and adaptive immune responses and can cause a febrile mononucleosis and hepatitis but is subclinical for most healthy individuals. Like the other herpesviruses, CMV is never fully cleared from the infected host and persists either in a true latent form or in a state of low-level replication made possible by multiple immune evasion mechanisms [7]. Reactivations from latency that can lead to virus transmission occur periodically and are triggered by various inflammatory stimuli and other physiologic stressors; however, they are largely asymptomatic and are mostly self-limited. Importantly, and unusually, immunity to CMV does not readily confer protection from super-infection, and secondary infection with other strains of CMV is not uncommon [8-11].

Active CMV infections can be identified by the presence of CMV nucleic acids and proteins in mucosal secretions. The frequency of CMV DNA shedding in the genital tract and saliva can vary substantially across different studies and is strongly dependent on the detection methods and cohort characteristics including geographical location [12, 13]. Such asymptomatic CMV shedding is important both for horizontal transmission and for the interactions of CMV with co-pathogens, adaptive immune responses, and innate immune activation. CMV is a large virus (approximately 236 kb) and is highly immunogenic—upwards of 10% of all memory T cells may be reactive to CMV antigens in persistently-infected individuals [14]—and many viral proteins have evolved to combat and evade host immunity [13]. When an infected individual has a compromised immune system, shedding of CMV DNA increases dramatically. In individuals who are profoundly immunocompromised (eg, AIDS or after transplant), CMV replication can be uncontrolled and lead to end-organ diseases such as pneumonitis, retinitis, hepatitis, or hemorrhagic colitis [7]. Congenital CMV infection is a substantial problem, particularly in resource-limited settings [15, 16], and can lead to hearing and vision loss, among other complications [15, 17-19].

While long thought to be relatively innocuous in immunocompetent adults, CMV infection is becoming more appreciated as an active contributor to a variety of complications. For example, in adults, CMV infection is associated with the development of cardiovascular diseases (CVD) including atherosclerosis, ischemic heart disease, and myocardial infarction, as well as cardiovascular death [20-22]. A recent meta-analysis of 10 prospective studies including over 30,000 participants determined exposure to CMV was associated with a 22% increased relative risk of developing CVD [20], and a comprehensive study of people with HIV infection (PWH) found that having anti-CMV antibodies (CMV seropositivity) was an independent risk factor for CVD and cerebrovascular diseases [23]. In this review, we discuss our current understanding of the mechanistic pathways by which CMV infection might promote CVD development in the general population, and, as a special case, in PWH. We also describe several innovative strategies to target CMV and how they might be applied to reducing CVD onset and severity.

## MECHANISTIC LINKS OF CMV TO CVD

### Systemic Inflammation

Persistent systemic inflammation is a risk factor for many complications, including CVD. Indeed, several systemic inflammatory mediators, such as high serum or plasma levels of interleukin (IL)-1β, IL-6, C-reactive protein (CRP), as well as tumor necrosis factor (TNF) receptor II (TNFR2), D-dimer, soluble CD14, and soluble CD163 have been shown to be associated with multiple cardiovascular outcomes and/or mortality [24-26]. Recently, in a large study of people at risk for CVD, treatment with the IL-1β antagonist canakinumab was shown to reduce systemic inflammation (ie, IL-6 and CRP levels) and also reduce the frequency of cardiovascular events even without lowering circulating lipid concentrations [27, 28]. IL-1β is produced by multiple cell types early after CMV infection or injury [29, 30]. While IL-1β can induce CMV reactivation from latency in cell culture and in mouse models [31, 32], evidence also suggests that CMV employs several mechanisms to counter IL-1β activity—potentially limiting its antiviral effects to allow for more robust infection [33-36].

IL-6 levels are highly correlated with CVD risk [37-39], and targeting IL-6 activity with tocilizumab has been shown to improve indices of endothelial dysfunction in participants at high risk for CVD [40, 41]. Acute CMV infection is associated with IL-6 expression both *in vitro* and *in vivo* [42-45]. During latency, plasma IL-6 levels remain elevated, even in those without active CMV replication, suggesting active CMV replication is not necessary for ongoing IL-6 expression [46, 47]. Accordingly, inhibiting CMV replication with ganciclovir in CMV seropositive critically ill individuals had no effect on IL-6 levels [47].

Pharmacologically targeting either IL-1β or IL-6 results in decreased CRP levels, consistent with CRP expression being downstream of their activities [27, 28, 48]. CRP can bind to low-density lipoproteins (LDL), allowing macrophages to take up the LDL without further modifications such as oxidation, and studies have shown increased inflammation following CRP treatment [49-51]. In a study of nearly 1000 participants with coronary artery disease, elevated CRP levels coupled with CMV seropositivity were associated with mortality, whereas neither elevated CRP levels without CMV nor CMV seropositivity without elevated CRP showed such an association [52]. These observations are consistent with other studies of the links among CMV, CRP, and CVD [53, 54].

TNF has been shown to be a major proinflammatory contributor to CVD risk in multiple studies, and targeting TNF can alleviate cardiovascular outcomes [55-57]. Much like for IL-1β, levels of TNF and CMV are associated [58], and TNF can induce CMV reactivation from cell lines [32, 58, 59]. However, CMV infection can impair TNF signaling *in vitro* [36, 60]. Thus, it is possible that while CMV limits TNF activity within infected cells to support its pathogenesis, continued TNF release from infected cells and from nearby cells during ongoing CMV replication leads to TNF accumulation in plasma [61]. Levels of the soluble TNF receptors TNFR1 and TNFR2 are surrogate markers of TNF activity *in vivo* [62], and their levels are linked to cardiovascular outcomes, including in PWH [25, 63]. As discussed in more detail below, we found that CMV infection was associated with elevated levels of soluble TNFR2 in PWH compared to CMV-uninfected PWH [64], and a small interventional study demonstrated that inhibiting CMV replication with valganciclovir resulted in a significant reduction in plasma levels of soluble TNFR2 in PWH [65]. Taken together, these data from multiple studies suggest that CMV infection (and likely continued low-level viral replication) is a critical determinant of persistent systemic inflammation, especially in those who are at risk of developing inflammation and CVD from other factors.

### Monocyte Activation

The stimulatory effect of CMV infection on the activity of immune cells has been well documented [66, 67]. Myeloid cells, including monocytes and macrophages, have long been known to be major contributors to CVD, and myeloid cells are some of the most abundant cells within plaque tissues [68, 69]. In atherosclerosis, monocytes enter the nascent plaque from the circulation where they encounter proinflammatory cytokines, take up oxidized lipoproteins via scavenger receptors, and differentiate into cholesterol-laden foam cells [70]. The combination of infiltration and cell retention results in macrophage accumulation in the plaques.

The latent CMV reservoir is retained mainly within CD34+ hematopoietic stem cells—including monocyte precursor cells—in the bone marrow [71-73]. Circulating monocytes harbor latent CMV that turns on viral gene expression upon differentiation into tissue macrophages [66]. The proinflammatory microenvironment in the developing plaque may thus induce CMV replication, because *in vitro* experiments have demonstrated that endothelial cell (EC) co-culture and oxidized LDL (oxLDL) can synergistically induce CMV gene expression [66]. CMV infection reciprocally promotes expression of the scavenger receptor CD36 and enhances oxLDL uptake in a monocyte/macrophage cell line [74]. CMV infection also increases production of IL-1β, IL-18, TNF, and interferon (IFN)-γ by infected monocytes and macrophages *in vitro* [61, 75]. These phenomena are of particular interest because of the proatherogenic effects of these cytokines in atherosclerosis. Mechanistically, CMV could promote early atherogenesis through direct effects on CMV-laden monocytes as they enter the nascent plaque, release proinflammatory cytokines, acquire oxidized lipoproteins, and differentiate into foam cells.

Intriguingly, CMV encodes several chemokine receptor homologs that induce signaling pathways in the infected cell. One of these is US28, a G-protein-coupled receptor that can bind to the chemokines CCL2, CCL5, CCL7, and CX3CL1, and which may contribute to the development of CMV-associated CVD [76, 77]. The binding of US28 to CX3CL1, also known as fractalkine, may be particularly important. CX3CL1 is abundantly expressed by vascular ECs, is upregulated by inflammation, is detected in atherosclerotic plaque tissues, and mediates both cell adhesion and chemotaxis of cells that express the fractalkine receptor (CX3CR1) [78, 79]. In addition, CX3CL1 binding to US28 induces macrophage migration [80]. As US28 expression is also necessary for the maintenance of CMV latency [81], circulating monocytes harboring latent CMV should have constitutive expression of US28. Thus, it is conceivable that latently infected cells might be more susceptible to the influence of CX3CL1 signals and traffic more readily to sites of endothelial dys-function, such as atherosclerotic plaques. As the infected monocyte is activated and differentiates, CMV could reactivate from its latent state, spread to surrounding ECs and smooth muscle cells, and further potentiate atherogenesis.

### T-cell Stimulation

Primary CMV infection drives expansion of CMV-specific CD4 and CD8 T cells. The functionality of these cells, and ultimately their capacity to prevent CMV recurrence, can be predicted by their expression of transcription factors, including T-bet and eomesodermin [82, 83]. Unlike many other viral infections in which the T-cell response is substantially reduced during the contraction phase and is maintained as a low-level memory response, latent CMV infection often drives the continued expansion of activated T cells, a process known as memory inflation. In humans, this phenomenon can result in a surprisingly large proportion of all memory T cells being specific for CMV antigens, upwards of 10% in the general population and even higher in PWH and the elderly [14, 84-86]. Although these CMV-specific T cells retain their functionality, and are not exhausted per se, they are incapable of either clearing CMV infection from the body or preventing CMV superinfections.

CD4 and CD8 T cells that express the fractalkine receptor (CX3CR1) have been associated with adverse cardiovascular outcomes [87-90]. As described above, fractalkine (CX3CL1) may drive the infiltration and retention of T cells, other CX3CR1+ cells (ie, macrophages and NK cells), and CMV-infected cells expressing US28 into the plaques where they may colocalize at sites of EC activation, interact, and contribute to atherogenesis [78-80]. We have recently shown that CMV infection is associated with an increase in the proportion of peripheral blood T cells that expresses CX3CR1 [91], and we and others have shown that CMV-specific CD8 T cells are enriched for CX3CR1 expression [91-94]. CX3CR1+ CD8 T cells are potent effector cells; they are enriched for the cytolytic enzymes granzyme B (GzB) and perforin [94, 95] and for production of IFN-γ and TNF following T-cell receptor (TCR)-mediated stimulation [93]. TNF release may be an important link between CMV-specific CX3CR1+ T cells and CVD: we have shown that TCR-activated CX3CR1+ CD8 T cells can induce the procoagulant tissue factor (TF) on the surface of monocytes in a TNF-dependent manner [96], and TF-expressing monocytes have been shown to promote coagulopathy in a non-human primate model of HIV infection [97]. Although less abundant than their CD8 T-cell counterparts, CX3CR1+ CD4 T cells are also enriched for TNF production [98-100], and our group recently demonstrated that CX3CR1+ CD4 T cells can migrate toward TNF-activated EC *in vitro* in a CX3CL1-dependent manner [101]. Accordingly, CMV-specific CD4 T cells in general [102], and CMV-specific CX3CR1+ CD4 T cells in particular [99], have been linked to increased carotid intima media thickness in PWH. Furthermore, T cells from CMV seropositive donors provoke endothelial damage, and EC upregulation of adhesive proteins and CX3CL1 [103, 104]. Thus, it is plausible that CMV infection results in an immune environment that is primed to exacerbate CVD via trafficking of CX3CR1+ T cells to sites of endothelial dysfunction, which are enriched in CX3CL1. The T cells can mediate further damage to the endothelium via cytokine and lytic granule release and interact with colocalized cells such as monocytes to initiate inflammatory and coagulation pathways. Whether these interactions and subsequent atherogenesis require specific recognition of CMV peptides is unclear, but evidence suggests that CMV nucleic acids and proteins are abundant in plaque tissues (see below). Following this, it is unclear if the cell-mediated mechanisms that may promote cardiovascular risk in CMV seropositive individuals are fundamentally different than those found in CMV seronegative people, or if they are just more abundant.

### CMV and Endothelium

The importance of the endothelium in the development and progression of atherosclerosis has long been recognized [105]. The condition of the endothelium determines the adhesive status of the vessel, vessel tone and permeability, the possibility for thrombus formation, and other features. The endothelial state can in turn correlate with the clinical complications of atherosclerosis [106-110]. Smoking, severe mental stress with high catecholamine emission, hemodynamic forces, oxLDL, IFNγ, TNF, and other factors can influence the health and function of the endothelium [111-116].

Numerous studies have revealed that CMV infection is a key determinant of endothelial status. Direct CMV infection causes vascular EC injury [117, 118], enhancing adhesiveness, and EC damage caused by CMV leads to the synthesis and expression of CX3CL1, intercellular adhesion molecule (ICAM)-1, vascular cell adhesion molecule (VCAM), von Willebrand factor (vWF), and other molecules [119-123]. CMV infection also results in EC apoptosis and downregulation of endothelial nitric oxide synthase (eNOS) expression [104, 119, 121, 122, 124], which may explain the experimentally impaired endothelium-dependent and endothelium-independent arterial vasodilation driven by CMV [125, 126]. Thus, CMV infection increases the permeability of the endothelium, the recruitment and trans-endothelial migration of monocytes [117], and possibly of CX3CR1+ T cells. CMV has recently been shown to affect the expression of matrix metalloproteinases in endothelial and smooth muscle cells, influencing atherosclerotic plaque stability [61, 75, 127]. Moreover, CMV infection-associated inflammation leads to an imbalance in the thrombogenic/antithrombogenic function of the endothelium, increasing platelet adhesion to the endothelium as well as the risk of thrombus formation [122, 128].

That CMV directly infects the vascular walls *in vivo* has been demonstrated in clinical studies of individuals suffering from inflammatory eye diseases [129]. CMV seropositivity has been linked to impaired vascular function, assessed from response to bradykinin [130] and brachial artery flow-mediated dilation (FMD) [131], but these results are somewhat controversial [132, 133]. In a study of children after heart transplantation, detectable CMV DNA in peripheral blood cells was associated with a significant impairment in FMD [134]. CMV infection in adult heart transplant recipients was associated with impaired endothelial function, assessed from coronary flow reserve [135]. These effects might be due in part to modulation of eNOS activity by CMV infection. Specifically, CMV infection results in an elevated level of asymmetric dimethylarginine (an endogenous eNOS inhibitor), which abolishes eNOS activation, in the serum of heart transplant patients [136]. Endothelial function was also assessed from angiography in coronary artery atherosclerosis patients with different titers of IgG antibodies against the coronary artery disease-related infections CMV, *Chlamydia pneumoniae, Helicobacter pylori,* hepatitis A virus, and herpes simplex virus (HSV)-1. Simultaneous detection of antibodies against several pathogens significantly correlated with deteriorated endothelium-dependent and endothelium-independent vasodilation in tests using adenosine and acetylcholine in both patients with atherosclerosis and volunteers [137]. In our recent study, we analyzed productive CMV infection using detection of CMV DNA in blood plasma of individuals with acute myocardial infarction and volunteers without CVD. We found a significantly higher frequency of presence and load of CMV DNA in patients with acute myocardial infarction than in controls [138]. Furthermore, when we assessed endothelial function in these patients, we for the first time showed a significant negative correlation between CMV DNA in plasma and the results of the FMD test in patients with acute myocardial infarction [139].

CMV infection is also closely related to increased production of the proinflammatory cytokine IL-15, which also activates T cells, enhances mitochondrial activity, and promotes the intracellular accumulation of cytolytic molecules such as GzB and perforin, as shown in our studies and in those of others [91, 101, 140-142]. In our recent report investigating the role of CMV infection on the development of CD57+ CD4 memory T cells, we found that IL-15 treatment *in vitro* upregulated intracellular GzB and perforin and surface expression of CX3CR1 by both CD57+ and CD57-CD4 memory T cells, but more so for CD57+ cells [101]. IL-15 has also been shown to promote the maintenance of CMV-specific inflationary memory T cells in a mouse model [142], and it has been linked to cardiovascular disease—serum IL-15 levels are elevated in patients with coronary artery disease, peripheral artery disease, and hypertension compared to controls [143, 144]. In addition, IL-15 is induced by a Western diet and exacerbates atherosclerotic lesion development in the LDL receptor knockout mouse model [145]. IL-15 protein is detected within atherosclerotic plaques in mice and humans, seemingly colocalized with macrophages [146, 147], and we recently showed that oxLDL exposure can trigger IL-15 production by monocytes *in vitro* [148]. Thus, IL-15 is a likely contributor to CVD, and the pathways that drive its expression in CMV seropositive individuals require further exploration.

As described above, CMV infection of the endothelium triggers active recruitment of peripheral mononuclear cells [117, 124], as well as the transfer of CMV antigens to T cells via exosome-like extracellular particles [149]. Therefore, it has been suggested that CMV infection of the endothelium results in secretion of viral proteins and mature viral particles, which are then presented by the neighboring ECs to smooth muscle cells [150]. As a result, chemokines actively recruit myeloid cells and T cells to the endothelium, leading to perpetuation of inflammation in the plaques and atherosclerosis progression. Extracellular vesicles (EVs) significantly increase in myocardial infarction [151]; this may be one of the mechanisms allowing CMV to spread among endothelial and immune cells. Over the last few years, the transfer of virus-derived genetic material between cells by EVs has been reported, which in some cases promoted further infection of the target cells with mature viral particles [152, 153] or even resulted in productive infection mediated by EVs [154, 155]. A similar mechanism has been examined in detail for the transmission of HIV, influenza virus, hepatitis B and C viruses, HSV-1, and HSV-2 [156], and recently the same pathway was demonstrated for CMV transfer in blood [150]. Furthermore, statins, which significantly decrease EV shedding in blood [157], can also suppress production of CMV DNA in cultured cells [158] and abolish the effect of CMV on the expansion of atherosclerosis in mice [159], supporting a role for EVs in CMV production and shedding.

## ANIMAL MODELS

Human CMV is highly species-specific, so current animal models of CMV infections and patho-physiology utilize related viruses. Indeed, much of the foundational animal work linking CMV and CVD has been performed in rat models. In a model of photochemically-induced femoral artery injury, rats infected with CMV at the time of injury or 2 weeks later exhibited increase media or neointima thickness, respectively, consistent with the interpretation that CMV infection stimulates cell proliferation in the vasculature [160]. Lemstrom and colleagues reported that CMV infection early after aortic allograft transplantation accelerated atherosclerosis development [161], which could be delayed by ganciclovir administration [162]. In a subsequent study, acute CMV infection was associated with significant EC proliferation and increased intimal thickening, and mononuclear cell infiltrates around CMV-infected cardiac allografts exhibited both early and late CMV antigen expression [163]. Murine CMV has also been shown to be associated with CVD—in fact, mice latently infected with CMV demonstrate vascular dysfunction, particularly as the mice age [126]—and atherosclerotic lesions in CMV-infected apolipoprotein E (ApoE) knockout mice are larger and more advanced than those in uninfected ApoE knockout controls [164]. Monkey studies of CMV pathogenesis, which might be most informative given the similarity to human physiology, are unfortunately hindered by the high prevalence of CMV infections in most non-human primate colonies.

## CMV AND HIV — A SPECIAL CASE

Although CMV and HIV are very different at the molecular level, both viruses persist lifelong, and almost all individuals infected with HIV are also infected with CMV (>90%). However, unlike CMV, which has been infecting humans since our species arose, HIV is a far younger virus, infecting humans for only the past hundred years or so, and currently an estimated 40 million people are living with HIV infection worldwide. In the absence of therapy, infection with HIV-1 results in a progressive loss of immune function marked by depletion of CD4 T cells, leading to opportunistic infections and malignancies characteristic of AIDS. Before the advent of antiretroviral therapy (ART), CMV disease was the most common viral complication of AIDS, leading to devastating blindness in many PWH. The introduction of ART in 1995–1996 profoundly reduced the incidence of AIDS-related CMV end-organ diseases [165]. Notably, the substantial overlap between the HIV epidemic and CMV prevalence does not appear to be coincidental. In addition to having similar risk factors for acquisition, accumulating data show that HIV and CMV infections enhance each other's pathogenesis not only by facilitating replication and transmission but also by exacerbating comorbidities, including CVD [12, 23].

In a large longitudinal study of PWH with (n=5119) or without (n=992) CMV coinfection, CMV seropositivity was linked to an increased risk of adverse clinical events, and the cardiovascular outcomes were the most frequent events associated with CMV [23]. HIV infection is also associated with elevated levels of systemic inflammatory mediators (such as IL-6 and interferon-inducible protein [IP]-10) and with chronic immune activation, as evidenced by persistently activated platelets, monocytes, and T cells [166]. The extent to which these effects are due to HIV infection, or are due to coinfections such as CMV, is not known. That CMV infection does contribute is clear however, as we and others have shown that CMV seronegative PWH lack the HIV-associated CD8 T-cell expansion [64, 167] and have significantly reduced plasma levels of IP-10, soluble TNFR2, and D-dimer compared to CMV seropositive PWH [64].

## STRATEGIES TO TARGET CMV

### Antiviral Drugs

Since CMV infection is a major complication in immunosuppressed individuals, targeting CMV with antivirals has been tried in several clinical settings, including in allogeneic hematopoietic stem cell transplant (HSCT) recipients and in PWH, but any cardiovascular benefits of these treatments are unclear. A randomized controlled study showed reductions in CMV shedding and in CD8 T-cell activation when PWH were treated for just 8 weeks with the anti-CMV drug valganciclovir [168]. Because this trial was small (n=30) and of relatively short duration, analysis of CVD outcomes was not possible, although soluble TNFR2 levels, which are associated with cardiovascular outcomes in PWH [25], were reduced following valganciclovir treatment [65]. Letermovir is a CMV DNA terminase complex inhibitor that is FDA-approved for prophylaxis of CMV infection and disease in adult CMV-seropositive allogeneic HSCT recipients [169-171]. Letermovir has superb antiviral activity against CMV with an excellent safety profile and little cross-resistance with other antivirals. Letermovir demonstrated significant benefit compared to placebo measured by time to clinically significant CMV disease post-HSCT (18.9% vs 44.3% cumulative rate; stratified log-rank test, 2-sided *P*-value <0.0001). Overall, letermovir has shown promising clinical efficacy and is generally well tolerated, thus providing a favorable new option in the prophylaxis of CMV infection and disease. Some limitations of letermovir are that the drug has no significant activity against other herpesvirus or non-human CMV, and it has a relatively low barrier for resistance. Our group is currently developing a clinical trial to test the safety and antiviral efficacy of letermovir in CMV-seropositive PWH. Although this trial will not be powered to detect if letermovir treatment reduces CVD, we plan to measure indices of arterial inflammation and levels of CVD risk biomarkers, including soluble TNFR2, which is strongly linked to cardiovascular outcomes and to CMV coinfection in PWH [25].

### Adoptive Cell Therapies

One of the most exciting areas of current research is the development of adoptive cell therapies. This term encompasses a wide range of clinical approaches that span from HSCT to the more recent developments of chimeric antigen receptor (CAR) T cells. In addition to its benefits for treating malignancies, HSCT is the only strategy that has resulted in functional cure of HIV infection [172, 173]. However, given the inherent dangers in the procedure and the lack of scalability, HSCT is unlikely to be a widely applied strategy for treatment of any viral infection, especially one as prevalent as CMV. In that sense, more directed approaches, such as CAR T and CAR NK cells, are more promising. CAR cells express engineered transmembrane constructs that contain the antigen-binding variable portion of an antibody extracellularly (to confer specificity) and the CD3ζ signaling portion of a TCR receptor intracellularly (to confer functionality), often with additional costimulatory elements included, thereby allowing the engineered cell to bypass traditional TCRMHC restrictions [174, 175]. CAR T cells that have specificity for CMV glycoprotein B (gB) have been developed and seem to limit CMV infection *in vitro* and in a humanized mouse model by cytokine release but not cytotoxicity [176-179]. Unfortunately, CAR T cells carry considerable risk, including that of cardiovascular complications [180, 181], and so it is unlikely anyone but the most vulnerable would be eligible for adoptive cell therapy with CMV-specific CAR T cells.

### Immunotherapy

Immunotherapy is another exciting area of advancement in cancer treatment that is being applied for infectious diseases. While the term “immunotherapy” refers to any agent that targets the host response as opposed to the tumor or pathogen (such as with chemotherapy or antivirals), most often the term is used to described therapies (usually antibodies) that inhibit components of immune checkpoint pathways. The FDA has approved immunotherapy regimens that target the immune checkpoint molecules programmed death (PD)-1, PD-ligand 1 (PD-L1), and cytotoxic T lymphocyte-associated protein 4 (CTLA-4), alone or in combination, and therapies that target other pathways are currently in development [182, 183]. Many CMV-reactive T cells express PD-1, although their functionality does not seem to be impaired [100, 184]. In general, these therapies inhibit the molecular pathways that actively limit T-cell function. Unfortunately, immunotherapy can also lead to considerable immune-related adverse events [185], and as with adoptive cell therapy, it is unlikely that immunotherapy against CMV will gain widespread use in healthy individuals. However, when someone who is CMV seropositive requires immunotherapy, it is possible that anti-CMV activity could be a welcome ‘side-effect’ of treatment, ie, PD-1 blockade could potentially license T cells to kill CMV-infected monocytes that they might otherwise ignore.

We should note that CMV encodes some of its own inhibitory agents that are potentially targets for immunotherapy. For instance, the viral UL18 protein is a homolog of class I major histocompatibility complex (MHC-I) molecules and can inhibit the function of cells that express the leukocyte Ig-like receptor, subfamily B (LILRB)-1 protein [186]. LILRB-1 expression is found on about half of the CMV-specific CD8 T cells, and blocking LILRB-1 activity increases IFN-γ production [187]. Since LILRB-1 binds UL18 with more than 1000-fold higher affinity than host MHC-I molecules [188], UL18 expressed by CMV-infected cells could be a potent inhibitor of immune responses *in vivo* [189]. Therefore, blocking LILRB-1/UL18 interactions might confer anti-CMV specificity with limited immunopathology and be a useful strategy to target CMV and its associated comorbidities.

### CMV Vaccines

The development of a CMV vaccine is a top priority due to its potential cost-effectiveness and associated public health benefits. Prophylactic vaccines might prevent the acquisition of CMV infection, thereby reducing overall infectious burden, and therapeutic vaccines might reduce reactivation from latency, virus shedding and transmission, and CMV-associated morbidities such as CVD. However, there are many challenges facing vaccine development including (1) complex immune evasion mechanisms, (2) unclear immune correlates of protection, and (3) a narrow range of CMV hosts which limits the value of animal models. In spite of these limitations, several types of CMV vaccine candidate, including live-attenuated, DNA, vectored, and peptide vaccines, have been developed or are currently under development, and have been reviewed in detail elsewhere [190, 191]. Recently, a modified vaccinia Ankara (MVA)-based vaccine (Triplex), which encodes 3 full-length CMV antigens—pp65 (UL83), IE1-exon4 (UL123), IE2-exon5 (UL122)—obtained excellent safety and immunostimulatory results in a phase 1 trial with healthy adults [192] and in a phase 2 trial of over 100 adult HSCT recipients at high risk for CMV reactivation [193]. A randomized placebo-controlled clinical trial to test the safety and efficacy of Triplex in PWH is being developed by our group. While the trial will not be powered to assess clinical outcomes such as a reduction in cardiovascular outcomes, we plan to measure whether vaccination elicits changes in plasma levels of soluble inflammatory markers associated with CVD.

### Cardiovascular Benefits of Anti-CMV Therapies

Most of the focus of anti-CMV therapies has understandably been to prevent acute congenital and transplant-associated infections, as these situations represent the most immediate need for interventions. However, given the association of CMV infection and cardiovascular risk, it is tempting to consider CMV as a target for novel strategies to treat and/or reduce CVD. The high prevalence of CMV within atherosclerotic plaques of patients undergoing endarterectomy [138, 194, 195], suggests that active CMV replication might be a contributor to plaque development and/or stability. Thus, these approaches could have a direct positive effect by protecting the vascular endothelium and smooth muscle cells that become infected with CMV. Alternatively, targeting CMV could have an indirect effect by reducing the proinflammatory milieu that is central to CVD risk. Of course, these outcomes are not mutually exclusive, and it may be a combination of these effects that ultimately proves protective. As CVD is already the leading cause of mortality worldwide [196], and expected to increase in prevalence [197], finding novel targets to alleviate disease burden is critical. Targeting CMV infection just might serve as such an opportunity.

## CMV AS VACCINE VECTOR

In addition to the advancements in anti-CMV drugs, vaccines, and other therapeutic approaches, another promising area of research has been to utilize CMV as a vaccine vector. This is an appealing strategy for a number of reasons, including its robust immunostimulatory potential, its lifelong persistence, and its ability to resist immune clearance in CMV-infected hosts [198, 199]. Indeed, preclinical studies have demonstrated that CMV vectors are highly efficacious against challenge with a wide array of pathogens including viruses (SIV), bacteria (*Mycobacterium tuberculosis),* parasites *(Plasmodium knowlesi*), and even cancers [200-207]. Current efforts are underway to translate this technology for safe usage in human trials [199, 206, 208, 209]. This potent vaccine efficacy seems to be linked to the development of vaccine antigen-specific effector memory CD8 T cells, as opposed to antibody or central memory T-cell responses [200, 210], a finding that is consistent with the important role of CD8 T-cell surveillance in limiting superinfections with CMV [211]. Interestingly, much of the vaccine efficacy seems to be due to the elicitation of CD8 T cells that recognize nonclassical MHC-E alleles [212], and how these observations will manifest in human studies remains to be seen. Nevertheless, caution must be taken to ensure that recipients of CMV-based vaccine constructs do not acquire increased risk of developing cardiovascular complications.

## CONCLUSION

Although traditional risk factors (smoking, diabetes, etc.) are certainly important drivers of CVD, it is becoming increasingly evident that persistent CMV infection is a contributor to a variety of cardiovascular complications, even in individuals without overt virus expression. Asymptomatic reactivation from latency is likely far more common than appreciated and promotes inflammation and activation of T cells and monocytes. Early in life, this response is possibly protective—evidence suggests latent CMV infection may promote influenza vaccine efficacy and clearance of bacterial coinfections [213, 214]—yet over time, this benefit is lost and the CMV-associated inflammation proves detrimental. When coupled with direct infection of vascular endothelial and smooth muscle cells, CMV becomes a key factor in cardiovascular pathogenesis. Given the high prevalence of latent CMV infection worldwide, innovative strategies to target the virus, like those described in this review, may be instrumental in helping to alleviate the tremendous burden of CVD.

## References

[1] Staras SA, Dollard SC, Radford KW, Flanders WD, Pass RF, Cannon MJ. Seroprevalence of cytomegalovirus infection in the United States, 1988-1994. Clinical infectious diseases : an official publication of the Infectious Diseases Society of America. 2006;43(9):1143-51. Epub 2006/10/10. doi: 10.1086/508173. PubMed PMID: 17029132. 17029132

[2] Bate SL, Dollard SC, Cannon MJ. Cytomegalovirus seroprevalence in the United States: the national health and nutrition examination surveys, 1988-2004. Clinical infectious diseases : an official publication of the Infectious Diseases Society of America. 2010;50(11):1439-47. Epub 2010/04/30. doi: 10.1086/652438. PubMed PMID: 20426575. 20426575 PMC11000537

[3] Griffiths PD, Baboonian C. A prospective study of primary cytomegalovirus infection during pregnancy: final report. Br J Obstet Gynaecol. 1984;91(4):307-15. Epub 1984/04/01. doi: 10.1111/j.1471-0528.1984.tb05915.x. PubMed PMID: 6324849. 6324849

[4] Tookey PA, Ades AE, Peckham CS. Cytomegalovirus prevalence in pregnant women: the influence of parity. Arch Dis Child. 1992;67(7 Spec No):779-83. Epub 1992/07/01. doi: 10.1136/adc.67.7_spec_no.779. PubMed PMID: 1325757; PMCID: PMC1590405. 1325757 PMC1590405

[5] Bates M, Brantsaeter AB. Human cytomegalovirus (CMV) in Africa: a neglected but important pathogen. J Virus Erad. 2016;2(3):136-42. Epub 2016/08/03. PubMed PMID: 27482452; PMCID: PMC4967964. 27482452 10.1016/S2055-6640(20)30456-8 PMC4967964

[6] Al Mana H, Yassine HM, Younes NN, Al-Mohannadi A, Al-Sadeq DW, Alhababi D, Nasser EA, Nasrallah GK. The Current Status of Cytomegalovirus (CMV) Prevalence in the MENA Region: A Systematic Review. Pathogens. 2019;8(4). Epub 2019/11/07. doi: 10.3390/pathogens8040213. PubMed PMID: 31683687; PMCID: PMC6963600. PMC6963600 31683687

[7] Griffiths P, Baraniak I, Reeves M. The pathogenesis of human cytomegalovirus. J Pathol. 2015;235(2):288-97. Epub 2014/09/11. doi: 10.1002/path.4437. PubMed PMID: 25205255. 25205255

[8] Spector SA, Hirata KK, Newman TR. Identification of multiple cytomegalovirus strains in homosexual men with acquired immunodeficiency syndrome. J Infect Dis. 1984;150(6):953-6. Epub 1984/12/01. doi: 10.1093/infdis/150.6.953. PubMed PMID: 6094679. 6094679

[9] Meyer-Konig U, Ebert K, Schrage B, Pollak S, Hufert FT. Simultaneous infection of healthy people with multiple human cytomegalovirus strains. Lancet. 1998;352(9136):1280-1. Epub 1998/10/27. doi: 10.1016/S0140-6736(05)70487-6. PubMed PMID: 9788461. 9788461

[10] Boppana SB, Rivera LB, Fowler KB, Mach M, Britt WJ. Intrauterine transmission of cytomegalovirus to infants of women with preconceptional immunity. N Engl J Med. 2001;344(18):1366-71. Epub 2001/05/03. doi: 10.1056/NEJM200105033441804. PubMed PMID: 11333993. 11333993

[11] Gorman S, Harvey NL, Moro D, Lloyd ML, Voigt V, Smith LM, Lawson MA, Shellam GR. Mixed infection with multiple strains of murine cytomegalovirus occurs following simultaneous or sequential infection of immunocompetent mice. J Gen Virol. 2006;87(Pt 5):1123-32. Epub 2006/04/11. doi: 10.1099/vir.0.81583-0. PubMed PMID: 16603512. 16603512

[12] Gianella S, Massanella M, Wertheim JO, Smith DM. The Sordid Affair Between Human Herpesvirus and HIV. J Infect Dis. 2015;212(6):845-52. Epub 2015/03/10. doi: 10.1093/infdis/jiv148. PubMed PMID: 25748324; PMCID: PMC4548466. 25748324 PMC4548466

[13] Freeman ML, Lederman MM, Gianella S. Partners in Crime: The Role of CMV in Immune Dysregulation and Clinical Outcome During HIV Infection. Current HIV/AIDS reports. 2016;13(1):10-9. Epub 2016/01/27. doi: 10.1007/s11904-016-0297-9. PubMed PMID: 26810437; PMCID: PMC5079703. 26810437 PMC5079703

[14] Sylwester AW, Mitchell BL, Edgar JB, Taormina C, Pelte C, Ruchti F, Sleath PR, Grabstein KH, Hosken NA, Kern F, Nelson JA, Picker LJ. Broadly targeted human cytomegalovirus-specific CD4+ and CD8+ T cells dominate the memory compartments of exposed subjects. The Journal of experimental medicine. 2005;202(5):673-85. Epub 2005/09/09. doi: 10.1084/jem.20050882. PubMed PMID: 16147978; PMCID: PMC2212883. 16147978 PMC2212883

[15] Kenneson A, Cannon MJ. Review and meta-analysis of the epidemiology of congenital cytomegalovirus (CMV) infection. Rev Med Virol. 2007;17(4):253-76. Epub 2007/06/21. doi: 10.1002/rmv.535. PubMed PMID: 17579921. 17579921

[16] Lanzieri TM, Dollard SC, Bialek SR, Grosse SD. Systematic review of the birth prevalence of congenital cytomegalovirus infection in developing countries. Int J Infect Dis. 2014;22:44-8. Epub 2014/03/19. doi: 10.1016/j.ijid.2013.12.010. PubMed PMID: 24631522; PMCID: PMC4829484. 24631522 PMC4829484

[17] Fowler KB, McCollister FP, Dahle AJ, Boppana S, Britt WJ, Pass RF. Progressive and fluctuating sensorineural hearing loss in children with asymptomatic congenital cytomegalovirus infection. J Pediatr. 1997;130(4):624-30. Epub 1997/04/01. doi: 10.1016/s0022-3476(97)70248-8. PubMed PMID: 9108862. 9108862

[18] Yamamoto AY, Mussi-Pinhata MM, Isaac Mde L, Amaral FR, Carvalheiro CG, Aragon DC, Manfredi AK, Boppana SB, Britt WJ. Congenital cytomegalovirus infection as a cause of sensorineural hearing loss in a highly immune population. Pediatr Infect Dis J. 2011;30(12):1043-6. Epub 2011/08/05. doi: 10.1097/INF.0b013e31822d9640. PubMed PMID: 21814153; PMCID: PMC3222783. 21814153 PMC3222783

[19] Manicklal S, Emery VC, Lazzarotto T, Boppana SB, Gupta RK. The “silent” global burden of congenital cytomegalovirus. Clin Microbiol Rev. 2013;26(1):86-102. Epub 2013/01/09. doi: 10.1128/CMR.00062-12. PubMed PMID: 23297260; PMCID: PMC3553672. 23297260 PMC3553672

[20] Wang H, Peng G, Bai J, He B, Huang K, Hu X, Liu D. Cytomegalovirus Infection and Relative Risk of Cardiovascular Disease (Ischemic Heart Disease, Stroke, and Cardiovascular Death): A Meta-Analysis of Prospective Studies Up to 2016. J Am Heart Assoc. 2017;6(7). Epub 2017/07/08. doi: 10.1161/JAHA.116.005025. PubMed PMID: 28684641; PMCID: PMC5586265. PMC5586265 28684641

[21] Lebedeva AM, Shpektor AV, Vasilieva EY, Margolis LB. Cytomegalovirus Infection in Cardiovascular Diseases. Biochemistry (Mosc). 2018;83(12):1437-47. Epub 2019/03/18. doi: 10.1134/S0006297918120027. PubMed PMID: 30878019. 30878019

[22] Du Y, Zhang G, Liu Z. Human cytomegalovirus infection and coronary heart disease: a systematic review. Virol J. 2018;15(1):31. Epub 2018/02/08. doi: 10.1186/s12985-018-0937-3. PubMed PMID: 29409508; PMCID: PMC5801777. 29409508 PMC5801777

[23] Lichtner M, Cicconi P, Vita S, Cozzi-Lepri A, Galli M, Lo Caputo S, Saracino A, De Luca A, Moioli M, Maggiolo F, Marchetti G, Vullo V, d'Arminio Monforte A, Study IF. Cytomegalovirus coinfection is associated with an increased risk of severe non-AIDS-defining events in a large cohort of HIV-infected patients. J Infect Dis. 2015;211(2):178-86. Epub 2014/08/02. doi: 10.1093/infdis/jiu417. PubMed PMID: 25081936. 25081936

[24] Kuller LH, Tracy R, Belloso W, De Wit S, Drummond F, Lane HC, Ledergerber B, Lundgren J, Neuhaus J, Nixon D, Paton NI, Neaton JD, Group ISS. Inflammatory and coagulation biomarkers and mortality in patients with HIV infection. PLoS medicine. 2008;5(10):e203. Epub 2008/10/24. doi: 10.1371/journal.pmed.0050203. PubMed PMID: 18942885; PMCID: PMC2570418. 18942885 PMC2570418

[25] Tenorio AR, Zheng Y, Bosch RJ, Krishnan S, Rodriguez B, Hunt PW, Plants J, Seth A, Wilson CC, Deeks SG, Lederman MM, Landay AL. Soluble markers of inflammation and coagulation but not T-cell activation predict non-AIDS-defining morbid events during suppressive antiretroviral treatment. J Infect Dis. 2014;210(8):1248-59. Epub 2014/05/06. doi: 10.1093/infdis/jiu254. PubMed PMID: 24795473; PMCID: PMC4192039. 24795473 PMC4192039

[26] Hunt PW, Sinclair E, Rodriguez B, Shive C, Clagett B, Funderburg N, Robinson J, Huang Y, Epling L, Martin JN, Deeks SG, Meinert CL, Van Natta ML, Jabs DA, Lederman MM. Gut epithelial barrier dysfunction and innate immune activation predict mortality in treated HIV infection. J Infect Dis. 2014;210(8):1228-38. Epub 2014/04/24. doi: 10.1093/infdis/jiu238. PubMed PMID: 24755434; PMCID: PMC4192038. 24755434 PMC4192038

[27] Ridker PM, Everett BM, Thuren T, MacFadyen JG, Chang WH, Ballantyne C, Fonseca F, Nicolau J, Koenig W, Anker SD, Kastelein JJP, Cornel JH, Pais P, Pella D, Genest J, Cifkova R, Lorenzatti A, Forster T, Kobalava Z, Vida-Simiti L, Flather M, Shimokawa H, Ogawa H, Dellborg M, Rossi PRF, Troquay RPT, Libby P, Glynn RJ, Group CT. Antiinflammatory Therapy with Canakinumab for Atherosclerotic Disease. N Engl J Med. 2017;377(12):1119-31. Epub 2017/08/29. doi: 10.1056/NEJMoa1707914. PubMed PMID: 28845751. 28845751

[28] Ridker PM, MacFadyen JG, Everett BM, Libby P, Thuren T, Glynn RJ, Group CT. Relationship of C-reactive protein reduction to cardiovascular event reduction following treatment with canakinumab: a secondary analysis from the CANTOS randomised controlled trial. Lancet. 2018;391(10118):319-28. Epub 2017/11/18. doi: 10.1016/S0140-6736(17)32814-3. PubMed PMID: 29146124. 29146124

[29] Woodroffe SB, Garnett HM, Danis VA. Interleukin-1 production and cell-activation response to cytomegalovirus infection of vascular endothelial cells. Arch Virol. 1993;133(3-4):295-308. Epub 1993/01/01. doi: 10.1007/BF01313770. PubMed PMID: 8257291. 8257291

[30] Dengler TJ, Raftery MJ, Werle M, Zimmermann R, Schonrich G. Cytomegalovirus infection of vascular cells induces expression of pro-inflammatory adhesion molecules by paracrine action of secreted interleukin-1beta. Transplantation. 2000;69(6):1160-8. Epub 2000/04/13. doi: 10.1097/00007890-200003270-00022. PubMed PMID: 10762222. 10762222

[31] Cook CH, Trgovcich J, Zimmerman PD, Zhang Y, Sedmak DD. Lipopolysaccha-ride, tumor necrosis factor alpha, or interleukin-1beta triggers reactivation of latent cytomegalovirus in immunocompetent mice. J Virol. 2006;80(18):9151-8. Epub 2006/08/31. doi: 10.1128/JVI.00216-06. PubMed PMID: 16940526; PMCID: PMC1563908. 16940526 PMC1563908

[32] O'Connor CM, Murphy EA. A myeloid progenitor cell line capable of supporting human cytomegalovirus latency and reactivation, resulting in infectious progeny. J Virol. 2012;86(18):9854-65. Epub 2012/07/05. doi: 10.1128/JVI.01278-12. PubMed PMID: 22761372; PMCID: PMC3446554. 22761372 PMC3446554

[33] Jarvis MA, Borton JA, Keech AM, Wong J, Britt WJ, Magun BE, Nelson JA. Human cytomegalovirus attenuates interleukin-1beta and tumor necrosis factor alpha proinflammatory signaling by inhibition of NF-kappaB activation. J Virol. 2006;80(11):5588-98. Epub 2006/05/16. doi: 10.1128/JVI.00060-06. PubMed PMID: 16699040; PMCID: PMC1472148. 16699040 PMC1472148

[34] Listman JA, Race JE, Walker-Kopp N, Unlu S, Auron PE. Inhibition of IL-1beta transcription by peptides derived from the hCMV IE2 transactivator. Mol Immunol. 2008;45(9):2667-77. Epub 2008/03/01. doi: 10.1016/j.molimm.2007.12.024. PubMed PMID: 18308397; PMCID: PMC2363159. 18308397 PMC2363159

[35] Botto S, Abraham J, Mizuno N, Pryke K, Gall B, Landais I, Streblow DN, Fruh KJ, DeFilippis VR. Human Cytomegalovirus Immediate Early 86-kDa Protein Blocks Transcription and Induces Degradation of the Immature Interleukin-1beta Protein during Virion-Mediated Activation of the AIM2 Inflammasome. mBio. 2019;10(1). Epub 2019/02/14. doi: 10.1128/mBio.02510-18. PubMed PMID: 30755509; PMCID: PMC6372796. PMC6372796 30755509

[36] Montag C, Wagner J, Gruska I, Hagemeier C. Human cytomegalovirus blocks tumor necrosis factor alpha- and interleukin-1beta-mediated NF-kappaB signaling. J Virol. 2006;80(23):11686-98. Epub 2006/09/29. doi: 10.1128/JVI.01168-06. PubMed PMID: 17005669; PMCID: PMC1642604. 17005669 PMC1642604

[37] Ridker PM, Rifai N, Stampfer MJ, Hennekens CH. Plasma concentration of inter-leukin-6 and the risk of future myocardial infarction among apparently healthy men. Circulation. 2000;101(15):1767-72. Epub 2000/04/19. doi: 10.1161/01.cir.101.15.1767. PubMed PMID: 10769275. 10769275

[38] Danesh J, Kaptoge S, Mann AG, Sarwar N, Wood A, Angleman SB, Wensley F, Higgins JP, Lennon L, Eiriksdottir G, Rumley A, Whincup PH, Lowe GD, Gudnason V. Long-term interleukin-6 levels and subsequent risk of coronary heart disease: two new prospective studies and a systematic review. PLoS medicine. 2008;5(4):e78. Epub 2008/04/11. doi: 10.1371/journal.pmed.0050078. PubMed PMID: 18399716; PMCID: PMC2288623. 18399716 PMC2288623

[39] Ridker PM, Luscher TF. Anti-inflammatory therapies for cardiovascular disease. Eur Heart J. 2014;35(27):1782-91. Epub 2014/05/28. doi: 10.1093/eurheartj/ehu203. PubMed PMID: 24864079; PMCID: PMC4155455. 24864079 PMC4155455

[40] Bacchiega BC, Bacchiega AB, Usnayo MJ, Bedirian R, Singh G, Pinheiro GD. Interleukin 6 Inhibition and Coronary Artery Disease in a High-Risk Population: A Prospective Community-Based Clinical Study. J Am Heart Assoc. 2017;6(3). Epub 2017/03/16. doi: 10.1161/JAHA.116.005038. PubMed PMID: 28288972; PMCID: PMC5524026. PMC5524026 28288972

[41] Ruiz-Limon P, Ortega R, Arias de la Rosa I, Abalos-Aguilera MDC, Perez-Sanchez C, Jimenez-Gomez Y, Peralbo-Santaella E, Font P, Ruiz-Vilches D, Ferrin G, Collantes-Estevez E, Escudero-Contreras A, Lopez-Pedrera C, Barbarroja N. Tocilizumab improves the proatherothrombotic profile of rheumatoid arthritis patients modulating endothelial dysfunction, NETosis, and inflammation. Transl Res. 2017;183:87-103. Epub 2016/12/29. doi: 10.1016/j.trsl.2016.12.003. PubMed PMID: 28027930. 28027930

[42] Iwamoto GK, Konicek SA. Cytomegalovirus immediate early genes upregulate interleukin-6 gene expression. Journal of investigative medicine : the official publication of the American Federation for Clinical Research. 1997;45(4):175-82. Epub 1997/04/01. PubMed PMID: 9154298. 9154298

[43] Humar A, St Louis P, Mazzulli T, McGeer A, Lipton J, Messner H, MacDonald KS. Elevated serum cytokines are associated with cytomegalovirus infection and disease in bone marrow transplant recipients. J Infect Dis. 1999;179(2):484-8. Epub 1999/01/07. doi: 10.1086/314602. PubMed PMID: 9878035. 9878035

[44] Blankenberg S, Rupprecht HJ, Bickel C, Espinola-Klein C, Rippin G, Hafner G, Ossendorf M, Steinhagen K, Meyer J. Cytomegalovirus infection with interleukin-6 response predicts cardiac mortality in patients with coronary artery disease. Circulation. 2001;103(24):2915-21. Epub 2001/06/20. doi: 10.1161/01.cir.103.24.2915. PubMed PMID: 11413080. 11413080

[45] Botto S, Streblow DN, DeFilippis V, White L, Kreklywich CN, Smith PP, Caposio P. IL-6 in human cytomegalovirus secretome promotes angiogenesis and survival of endothelial cells through the stimulation of survivin. Blood. 2011;117(1):352-61. Epub 2010/10/12. doi: 10.1182/blood-2010-06-291245. PubMed PMID: 20930069; PMCID: PMC3037756. 20930069 PMC3037756

[46] Tong CY, Bakran A, Williams H, Cuevas LE, Peiris JS, Hart CA. Association of tumour necrosis factor alpha and interleukin 6 levels with cytomegalovirus DNA detection and disease after renal transplantation. J Med Virol. 2001;64(1):29-34. Epub 2001/04/04. doi: 10.1002/jmv.1013. PubMed PMID: 11285565. 11285565

[47] Limaye AP, Stapleton RD, Peng L, Gunn SR, Kimball LE, Hyzy R, Exline MC, Files DC, Morris PE, Frankel SK, Mikkelsen ME, Hite D, Enfield KB, Steingrub J, O'Brien J, Parsons PE, Cuschieri J, Wunderink RG, Hotchkin DL, Chen YQ, Ruben-feld GD, Boeckh M. Effect of Ganciclovir on IL-6 Levels Among Cytomegalovirus-Seropositive Adults With Critical Illness: A Randomized Clinical Trial. JAMA. 2017;318(8):731-40. Epub 2017/08/23. doi: 10.1001/jama.2017.10569. PubMed PMID: 28829877; PMCID: PMC5817487. 28829877 PMC5817487

[48] Rodriguez B, Chen Z, Tatsuoka C, Sieg SF, Landay A, McComsey GA, Clagett B, Longenecker CT, Shive C, Crawford KW, Moisi D, Freeman ML, Funderburg N, Calabrese L, Lederman MM. IL-6 blockade decreases inflammation and increases CD127 expression in HIV infection. Conference on Retroviruses and Opportunistic Infections; Boston, MA: IAS-USA; 2020.

[49] Pasceri V, Willerson JT, Yeh ET. Direct proinflammatory effect of C-reactive protein on human endothelial cells. Circulation. 2000;102(18):2165-8. Epub 2000/11/01. doi: 10.1161/01.cir.102.18.2165. PubMed PMID: 11056086. 11056086

[50] Zwaka TP, Hombach V, Torzewski J. C-reactive protein-mediated low density lipoprotein uptake by macrophages: implications for atherosclerosis. Circulation. 2001;103(9):1194-7. Epub 2001/03/10. doi: 10.1161/01.cir.103.9.1194. PubMed PMID: 11238260. 11238260

[51] Bisoendial RJ, Kastelein JJ, Levels JH, Zwaginga JJ, van den Bogaard B, Reitsma PH, Meijers JC, Hartman D, Levi M, Stroes ES. Activation of inflammation and coagulation after infusion of C-reactive protein in humans. Circ Res. 2005;96(7):714-6. Epub 2005/03/19. doi: 10.1161/01.RES.0000163015.67711.AB. PubMed PMID: 15774855. 15774855

[52] Muhlestein JB, Horne BD, Carlquist JF, Madsen TE, Bair TL, Pearson RR, Anderson JL. Cytomegalovirus seropositivity and C-reactive protein have independent and combined predictive value for mortality in patients with angiographically demonstrated coronary artery disease. Circulation. 2000;102(16):1917-23. Epub 2000/10/18. doi: 10.1161/01.cir.102.16.1917. PubMed PMID: 11034939. 11034939

[53] Zhu J, Quyyumi AA, Norman JE, Csako G, Epstein SE. Cytomegalovirus in the pathogenesis of atherosclerosis: the role of inflammation as reflected by elevated C-reactive protein levels. J Am Coll Cardiol. 1999;34(6):1738-43. Epub 1999/11/30. doi: 10.1016/s0735-1097(99)00410-6. PubMed PMID: 10577564. 10577564

[54] Zhu J, Shearer GM, Norman JE, Pinto LA, Marincola FM, Prasad A, Waclawiw MA, Csako G, Quyyumi AA, Epstein SE. Host response to cytomegalovirus infection as a determinant of susceptibility to coronary artery disease: sex-based differences in inflammation and type of immune response. Circulation. 2000;102(20):2491-6. Epub 2000/11/15. doi: 10.1161/01.cir.102.20.2491. PubMed PMID: 11076822. 11076822

[55] Ferrari R. The role of TNF in cardiovascular disease. Pharmacol Res. 1999;40(2):97-105. Epub 1999/08/06. doi: 10.1006/phrs.1998.0463. PubMed PMID: 10433867. 10433867

[56] Van Taunay JS, Albelda MT, Frias JC, Lipinski MJ. Biologics and Cardiovascular Disease. J Cardiovasc Pharmacol. 2018;72(2):77-85. Epub 2018/05/09. doi: 10.1097/FJC.0000000000000595. PubMed PMID: 29738370. 29738370

[57] Williams JW, Huang LH, Randolph GJ. Cytokine Circuits in Cardiovascular Disease. Immunity. 2019;50(4):941-54. Epub 2019/04/18. doi: 10.1016/j.immuni.2019.03.007. PubMed PMID: 30995508; PMCID: PMC6924925. 30995508 PMC6924925

[58] Docke WD, Prosch S, Fietze E, Kimel V, Zuckermann H, Klug C, Syrbe U, Kruger DH, von Baehr R, Volk HD. Cytomegalovirus reactivation and tumour necrosis factor. Lancet. 1994;343(8892):268-9. Epub 1994/01/29. doi: 10.1016/s0140-6736(94)91116-9. PubMed PMID: 7905100. 7905100

[59] Forte E, Swaminathan S, Schroeder MW, Kim JY, Terhune SS, Hummel M. Tumor Necrosis Factor Alpha Induces Reactivation of Human Cytomegalovirus Independently of Myeloid Cell Differentiation following Posttranscriptional Establishment of Latency. mBio. 2018;9(5). Epub 2018/09/13. doi: 10.1128/mBio.01560-18. PubMed PMID: 30206173; PMCID: PMC6134100. PMC6134100 30206173

[60] Baillie J, Sahlender DA, Sinclair JH. Human cytomegalovirus infection inhibits tumor necrosis factor alpha (TNF-alpha) signaling by targeting the 55-kilodalton TNF-alpha receptor. J Virol. 2003;77(12):7007-16. Epub 2003/05/28. doi: 10.1128/jvi.77.12.7007-7016.2003. PubMed PMID: 12768019; PMCID: PMC156201. 12768019 PMC156201

[61] Smith PD, Saini SS, Raffeld M, Manischewitz JF, Wahl SM. Cytomegalovirus induction of tumor necrosis factor-alpha by human monocytes and mucosal macrophages. J Clin Invest. 1992;90(5):1642-8. Epub 1992/11/01. doi: 10.1172/JCI116035. PubMed PMID: 1331170; PMCID: PMC443219. 1331170 PMC443219

[62] Valle Y, Ledezma-Lozano IY, Torres-Carrillo N, Padilla-Gutierrez JR, Navarro-Hernandez RE, Vazquez-Del Mercado M, Palafox-Sanchez CA, Armendariz-Borunda J, Munoz-Valle JF. Circulating TNFRI and TNFRII levels correlated with the disease activity score (DAS28) in rheumatoid arthritis. Scandinavian journal of rheumatology. 2009;38(5):332-5. Epub 2009/07/07. doi: 10.1080/03009740902865456. PubMed PMID: 19579138. 19579138

[63] Hoenigl M, Moser CB, Funderburg N, Bosch R, Kantor A, Zhang Y, Eugen-Olsen J, Finkelman M, Reiser J, Landay A, Moisi D, Lederman MM, Gianella S, Adult Clinical Trials Group Nst Soluble Urokinase Plasminogen Activator Receptor Is Predictive of Non-AIDS Events During Antiretroviral Therapy-mediated Viral Suppression. Clinical infectious diseases : an official publication of the Infectious Diseases Society of America. 2019;69(4):676-86. Epub 2018/11/13. doi: 10.1093/cid/ciy966. PubMed PMID: 30418519; PMCID: PMC6669298. 30418519 PMC6669298

[64] Freeman ML, Mudd JC, Shive CL, Younes SA, Panigrahi S, Sieg SF, Lee SA, Hunt PW, Calabrese LH, Gianella S, Rodriguez B, Lederman MM. CD8 T-Cell Expansion and Inflammation Linked to CMV Coinfection in ART-treated HIV Infection. Clinical infectious diseases : an official publication of the Infectious Diseases Society of America. 2016;62(3):392-6. Epub 2015/09/25. doi: 10.1093/cid/civ840. PubMed PMID: 26400999; PMCID: PMC4706630. 26400999 PMC4706630

[65] Beck-Engeser GB, Maldarelli F, York VA, Deeks SG, Martin JN, SInclair E, Hsue P, Tracy R, Huang Y, Hunt PW. Valganciclovir reduces sTNF-R2 and vascular dysfunction markers in treated HIV. Conference on Retroviruses and Opportunistic Infections; Seattle, WA: IAS-USA; 2019.

[66] Guetta E, Guetta V, Shibutani T, Epstein SE. Monocytes harboring cytomegalovirus: interactions with endothelial cells, smooth muscle cells, and oxidized low-density lipoprotein. Possible mechanisms for activating virus delivered by monocytes to sites of vascular injury. Circ Res. 1997;81(1):8-16. Epub 1997/07/01. doi: 10.1161/01.res.81.1.8. PubMed PMID: 9201022. 9201022

[67] Skarman PJ, Rahbar A, Xie X, Soderberg-Naucler C. Induction of polymorphonuclear leukocyte response by human cytomegalovirus. Microbes Infect. 2006;8(6):1592-601. Epub 2006/05/17. doi: 10.1016/j.micinf.2006.01.017. PubMed PMID: 16702012. 16702012

[68] Cochain C, Vafadarnejad E, Arampatzi P, Pelisek J, Winkels H, Ley K, Wolf D, Saliba AE, Zernecke A. Single-Cell RNA-Seq Reveals the Transcriptional Landscape and Heterogeneity of Aortic Macrophages in Murine Atherosclerosis. Circ Res. 2018;122(12):1661-74. Epub 2018/03/17. doi: 10.1161/CIRCRESAHA.117.312509. PubMed PMID: 29545365. 29545365

[69] Winkels H, Ehinger E, Vassallo M, Buscher K, Dinh HQ, Kobiyama K, Hamers AAJ, Cochain C, Vafadarnejad E, Saliba AE, Zernecke A, Pramod AB, Ghosh AK, Anto Michel N, Hoppe N, Hilgendorf I, Zirlik A, Hedrick CC, Ley K, Wolf D. Atlas of the Immune Cell Repertoire in Mouse Atherosclerosis Defined by Single-Cell RNA-Sequencing and Mass Cytometry. Circ Res. 2018;122(12):1675-88. Epub 2018/03/17. doi: 10.1161/CIRCRESAHA.117.312513. PubMed PMID: 29545366; PMCID: PMC5993603. 29545366 PMC5993603

[70] Moore KJ, Koplev S, Fisher EA, Tabas I, Bjorkegren JLM, Doran AC, Kovacic JC. Macrophage Trafficking, Inflammatory Resolution, and Genomics in Atherosclerosis: JACC Macrophage in CVD Series (Part 2). J Am Coll Cardiol. 2018;72(18):2181-97. Epub 2018/10/27. doi: 10.1016/j.jacc.2018.08.2147. PubMed PMID: 30360827; PMCID: PMC6522246. 30360827 PMC6522246

[71] Maciejewski JP, Bruening EE, Donahue RE, Mocarski ES, Young NS, Jeor SC. St Infection of hematopoietic progenitor cells by human cytomegalovirus. Blood. 1992;80(1):170-8. Epub 1992/07/01. PubMed PMID: 1377049. 1377049

[72] Kondo K, Kaneshima H, Mocarski ES. Human cytomegalovirus latent infection of granulocyte-macrophage progenitors. Proc Natl Acad Sci U S A. 1994;91(25):11879-83. Epub 1994/12/06. doi: 10.1073/pnas.91.25.11879. PubMed PMID: 7991550; PMCID: PMC45339. 7991550 PMC45339

[73] Minton EJ, Tysoe C, Sinclair JH, Sissons JG. Human cytomegalovirus infection of the monocyte/macrophage lineage in bone marrow. J Virol. 1994;68(6):4017-21. Epub 1994/06/01. doi: 10.1128/JVI.68.6.4017-4021.1994. PubMed PMID: 8189535; PMCID: PMC236908. 8189535 PMC236908

[74] Carlquist JF, Muhlestein JB, Horne BD, Hart NI, Lim T, Habashi J, Anderson JG, Anderson JL. Cytomegalovirus stimulated mRNA accumulation and cell surface expression of the oxidized LDL scavenger receptor, CD36. Atherosclerosis. 2004;177(1):53-9. Epub 2004/10/19. doi: 10.1016/j.atherosclerosis.2004.07.010. PubMed PMID: 15488865. 15488865

[75] Vliegen I, Duijvestijn A, Stassen F, Bruggeman C. Murine cytomegalovirus infection directs macrophage differentiation into a pro-inflammatory immune phenotype: implications for atherogenesis. Microbes Infect. 2004;6(12):1056-62. Epub 2004/09/24. doi: 10.1016/j.micinf.2004.05.020. PubMed PMID: 15380774. 15380774

[76] Streblow DN, Orloff SL, Nelson JA. The HCMV chemokine receptor US28 is a potential target in vascular disease. Curr Drug Targets Infect Disord. 2001;1(2):151-8. Epub 2002/11/29. doi: 10.2174/1568005014606080. PubMed PMID: 12455411. 12455411

[77] Vomaske J, Nelson JA, Streblow DN. Human Cytomegalovirus US28: a functionally selective chemokine binding receptor. Infect Disord Drug Targets. 2009;9(5):548-56. Epub 2009/07/15. doi: 10.2174/187152609789105696. PubMed PMID: 19594424; PMCID: PMC3496389. 19594424 PMC3496389

[78] Liu H, Jiang D. Fractalkine/CX3CR1 and atherosclerosis. Clinica chimica acta; international journal of clinical chemistry. 2011;412(13-14):1180-6. Epub 2011/04/16. doi: 10.1016/j.cca.2011.03.036. PubMed PMID: 21492740. 21492740

[79] Shin MS, You S, Kang Y, Lee N, Yoo SA, Park K, Kang KS, Kim SH, Mohanty S, Shaw AC, Montgomery RR, Hwang D, Kang I. DNA Methylation Regulates the Differential Expression of CX3CR1 on Human IL-7Ralphalow and IL-7Ralphahigh Effector Memory CD8+ T Cells with Distinct Migratory Capacities to the Fractalkine. Journal of immunology. 2015;195(6):2861-9. Epub 2015/08/16. doi: 10.4049/jimmunol.1500877. PubMed PMID: 26276874; PMCID: PMC4561204. PMC4561204 26276874

[80] Vomaske J, Melnychuk RM, Smith PP, Powell J, Hall L, DeFilippis V, Fruh K, Smit M, Schlaepfer DD, Nelson JA, Streblow DN. Differential ligand binding to a human cytomegalovirus chemokine receptor determines cell type-specific motility. PLoS Pathog. 2009;5(2):e1000304. Epub 2009/02/21. doi: 10.1371/journal.ppat.1000304. PubMed PMID: 19229316; PMCID: PMC2637432. 19229316 PMC2637432

[81] Krishna BA, Humby MS, Miller WE, O'Connor CM. Human cytomegalovirus G protein-coupled receptor US28 promotes latency by attenuating c-fos. Proc Natl Acad Sci U S A. 2019;116(5):1755-64. Epub 2019/01/17. doi: 10.1073/pnas.1816933116. PubMed PMID: 30647114; PMCID: PMC6358704. 30647114 PMC6358704

[82] Popescu I, Pipeling MR, Shah PD, Orens JB, McDyer JF. T-bet:Eomes balance, effector function, and proliferation of cytomegalovirus-specific CD8+ T cells during primary infection differentiates the capacity for durable immune control. Journal of immunology. 2014;193(11):5709-22. Epub 2014/10/24. doi: 10.4049/jimmunol.1401436. PubMed PMID: 25339676; PMCID: PMC4239205. PMC4239205 25339676

[83] Hoji A, Popescu ID, Pipeling MR, Shah PD, Winters SA, McDyer JF. Early KLRG1(+) but Not CD57(+)CD8(+) T Cells in Primary Cytomegalovirus Infection Predict Effector Function and Viral Control. Journal of immunology. 2019;203(8):2063-75. Epub 2019/09/27. doi: 10.4049/jimmunol.1900399. PubMed PMID: 31554693; PMCID: PMC7081945. PMC7081945 31554693

[84] Khan N, Shariff N, Cobbold M, Bruton R, Ainsworth JA, Sinclair AJ, Nayak L, Moss PA. Cytomegalovirus seropositivity drives the CD8 T cell repertoire toward greater clonality in healthy elderly individuals. Journal of immunology. 2002;169(4):1984-92. Epub 2002/08/08. doi: 10.4049/jimmunol.169.4.1984. PubMed PMID: 12165524. 12165524

[85] Stone SF, Price P, Khan N, Moss PA, French MA. HIV patients on antiretroviral therapy have high frequencies of CD8 T cells specific for Immediate Early protein-1 of cytomegalovirus. Aids. 2005;19(6):555-62. Epub 2005/04/02. doi: 10.1097/01.aids.0000163931.68907.7e. PubMed PMID: 15802973. 15802973

[86] Naeger DM, Martin JN, Sinclair E, Hunt PW, Bangsberg DR, Hecht F, Hsue P, McCune JM, Deeks SG. Cytomegalovirus-specific T cells persist at very high levels during long-term antiretroviral treatment of HIV disease. PLoS One. 2010;5(1):e8886. Epub 2010/02/04. doi: 10.1371/journal.pone.0008886. PubMed PMID: 20126452; PMCID: PMC2813282. 20126452 PMC2813282

[87] Ikejima H, Imanishi T, Tsujioka H, Kashiwagi M, Kuroi A, Tanimoto T, Kitabata H, Ishibashi K, Komukai K, Takeshita T, Akasaka T. Upregulation of fractalkine and its receptor, CX3CR1, is associated with coronary plaque rupture in patients with unstable angina pectoris. Circ J. 2010;74(2):337-45. Epub 2009/12/19. doi: 10.1253/circj.cj-09-0484. PubMed PMID: 20019415. 20019415

[88] Kasama T, Wakabayashi K, Sato M, Takahashi R, Isozaki T. Relevance of the CX-3CL1/fractalkine-CX3CR1 pathway in vasculitis and vasculopathy. Transl Res. 2010;155(1):20-6. Epub 2009/12/17. doi: 10.1016/j.trsl.2009.08.009. PubMed PMID: 20004358. 20004358

[89] Boag SE, Das R, Shmeleva EV, Bagnall A, Egred M, Howard N, Bennaceur K, Zaman A, Keavney B, Spyridopoulos I. T lymphocytes and fractalkine contribute to myocardial ischemia/reperfusion injury in patients. J Clin Invest. 2015;125(8):3063-76. Epub 2015/07/15. doi: 10.1172/JCI80055. PubMed PMID: 26168217; PMCID: PMC4563749. 26168217 PMC4563749

[90] Dong L, Nordlohne J, Ge S, Hertel B, Melk A, Rong S, Haller H, von Vietinghoff S. T Cell CX3CR1 Mediates Excess Atherosclerotic Inflammation in Renal Impairment. J Am Soc Nephrol. 2016;27(6):1753-64. Epub 2015/10/10. doi: 10.1681/ASN.2015050540. PubMed PMID: 26449606; PMCID: PMC4884117. 26449606 PMC4884117

[91] Morris SR, Chen B, Mudd JC, Panigrahi S, Shive CL, Sieg SF, Cameron CM, Zidar DA, Funderburg NT, Younes SA, Rodriguez B, Gianella S, Lederman MM, Freeman ML. Inflammescent CX3CR1+CD57+CD8+ T cells are generated and expanded by IL-15. JCI Insight. 2020;5(11). Epub 2020/05/06. doi: 10.1172/jci.insight.132963. PubMed PMID: 32369455; PMCID: PMC7346586. PMC7346586 32369455

[92] Gordon CL, Lee LN, Swadling L, Hutchings C, Zinser M, Highton AJ, Capone S, Folgori A, Barnes E, Klenerman P. Induction and Maintenance of CX3CR1-Intermediate Peripheral Memory CD8(+) T Cells by Persistent Viruses and Vaccines. Cell Rep. 2018;23(3):768-82. Epub 2018/04/19. doi: 10.1016/j.celrep.2018.03.074. PubMed PMID: 29669283; PMCID: PMC5917822. 29669283 PMC5917822

[93] Mudd JC, Panigrahi S, Kyi B, Moon SH, Manion MM, Younes SA, Sieg SF, Funderburg NT, Zidar DA, Lederman MM, Freeman ML. Inflammatory Function of CX3CR1+ CD8+ T Cells in Treated HIV Infection Is Modulated by Platelet Interactions. J Infect Dis. 2016;214(12):1808-16. Epub 2016/10/06. doi: 10.1093/infdis/jiw463. PubMed PMID: 27703039; PMCID: PMC5142088. 27703039 PMC5142088

[94] Bottcher JP, Beyer M, Meissner F, Abdullah Z, Sander J, Hochst B, Eickhoff S, Rieckmann JC, Russo C, Bauer T, Flecken T, Giesen D, Engel D, Jung S, Busch DH, Protzer U, Thimme R, Mann M, Kurts C, Schultze JL, Kastenmuller W, Knolle PA. Functional classification of memory CD8(+) T cells by CX3CR1 expression. Nature communications. 2015;6:8306. Epub 2015/09/26. doi: 10.1038/ncomms9306. PubMed PMID: 26404698; PMCID: PMC4667439. PMC4667439 26404698

[95] Nishimura M, Umehara H, Nakayama T, Yoneda O, Hieshima K, Kakizaki M, Dohmae N, Yoshie O, Imai T. Dual functions of fractalkine/CX3C ligand 1 in trafficking of perforin+/granzyme B+ cytotoxic effector lymphocytes that are defined by CX-3CR1 expression. Journal of immunology. 2002;168(12):6173-80. Epub 2002/06/11. doi: 10.4049/jimmunol.168.12.6173. PubMed PMID: 12055230. 12055230

[96] Freeman ML, Panigrahi S, Chen B, Juchnowski S, Sieg SF, Lederman MM, Funderburg NT, Zidar DA. CD8+ T-Cell-Derived Tumor Necrosis Factor Can Induce Tissue Factor Expression on Monocytes. J Infect Dis. 2019;220(1):73-7. Epub 2019/01/31. doi: 10.1093/infdis/jiz051. PubMed PMID: 30698729; PMCID: PMC6548895. 30698729 PMC6548895

[97] Schechter ME, Andrade BB, He T, Richter GH, Tosh KW, Policicchio BB, Singh A, Raehtz KD, Sheikh V, Ma D, Brocca-Cofano E, Apetrei C, Tracy R, Ribeiro RM, Sher A, Francischetti IMB, Pandrea I, Sereti I. Inflammatory monocytes expressing tissue factor drive SIV and HIV coagulopathy. Science translational medicine. 2017;9(405). Epub 2017/09/01. doi: 10.1126/scitranslmed.aam5441. PubMed PMID: 28855397; PMCID: PMC5755598. PMC5755598 28855397

[98] Kobayashi T, Okamoto S, Iwakami Y, Nakazawa A, Hisamatsu T, Chinen H, Kamada N, Imai T, Goto H, Hibi T. Exclusive increase of CX3CR1+CD28-CD4+ T cells in inflammatory bowel disease and their recruitment as intraepithelial lymphocytes. Inflamm Bowel Dis. 2007;13(7):837-46. Epub 2007/02/08. doi: 10.1002/ibd.20113. PubMed PMID: 17285595. 17285595

[99] Sacre K, Hunt PW, Hsue PY, Maidji E, Martin JN, Deeks SG, Autran B, McCune JM. A role for cytomegalovirus-specific CD4+CX3CR1+ T cells and cytomegalovirus-induced T-cell immunopathology in HIV-associated atherosclerosis. Aids. 2012;26(7):805-14. Epub 2012/02/09. doi: 10.1097/QAD.0b013e328351f780. PubMed PMID: 22313962; PMCID: PMC4155398. 22313962 PMC4155398

[100] Pachnio A, Ciaurriz M, Begum J, Lal N, Zuo J, Beggs A, Moss P. Cytomegalovirus Infection Leads to Development of High Frequencies of Cytotoxic Virus-Specific CD4+ T Cells Targeted to Vascular Endothelium. PLoS Pathog. 2016;12(9):e1005832. Epub 2016/09/09. doi: 10.1371/journal.ppat.1005832. PubMed PMID: 27606804; PMCID: PMC5015996. 27606804 PMC5015996

[101] Chen B, Morris SR, Panigrahi S, Michaelson GM, Wyrick JM, Komissarov AA, Potashnikova D, Lebedeva A, Younes SA, Harth K, Kashyap VS, Vasilieva E, Margolis L, Zidar DA, Sieg SF, Shive CL, Funderburg NT, Gianella S, Lederman MM, Freeman ML. Cytomegalovirus Coinfection Is Associated with Increased Vascular-Homing CD57(+) CD4 T Cells in HIV Infection. Journal of immunology. 2020;204(10):2722-33. Epub 2020/04/02. doi: 10.4049/jimmunol.1900734. PubMed PMID: 32229536; PMCID: PMC7315224. PMC7315224 32229536

[102] Hsue PY, Hunt PW, Sinclair E, Bredt B, Franklin A, Killian M, Hoh R, Martin JN, McCune JM, Waters DD, Deeks SG. Increased carotid intima-media thickness in HIV patients is associated with increased cytomegalovirus-specific T-cell responses. Aids. 2006;20(18):2275-83. Epub 2006/11/23. doi: 10.1097/QAD.0b013e3280108704. PubMed PMID: 17117013. 17117013

[103] Bolovan-Fritts CA, Spector SA. Endothelial damage from cytomegalovirus-specific host immune response can be prevented by targeted disruption of fractalkine-CX-3CR1 interaction. Blood. 2008;111(1):175-82. Epub 2007/09/27. doi: 10.1182/blood-2007-08-107730. PubMed PMID: 17895402; PMCID: PMC2200803. 17895402 PMC2200803

[104] van de Berg PJ, Yong SL, Remmerswaal EB, van Lier RA, ten Berge IJ. Cytomegalo-virus-induced effector T cells cause endothelial cell damage. Clin Vaccine Immunol. 2012;19(5):772-9. Epub 2012/03/09. doi: 10.1128/CVI.00011-12. PubMed PMID: 22398244; PMCID: PMC3346330. 22398244 PMC3346330

[105] Ross R, Harker L. Platelets, endothelium, and smooth muscle cells in atherosclerosis. Adv Exp Med Biol. 1978;102:135-41. Epub 1978/01/01. doi: 10.1007/978-1-4757-1217-9_8. PubMed PMID: 356557. 356557

[106] Halcox JP, Schenke WH, Zalos G, Mincemoyer R, Prasad A, Waclawiw MA, Nour KR, Quyyumi AA. Prognostic value of coronary vascular endothelial dys-function. Circulation. 2002;106(6):653-8. Epub 2002/08/07. doi: 10.1161/01.cir.0000025404.78001.d8. PubMed PMID: 12163423. 12163423

[107] Huang PH, Chen JW, Lu TM, Yu-An Ding P, Lin SJ. Combined use of endothelial function assessed by brachial ultrasound and high-sensitive C-reactive protein in predicting cardiovascular events. Clin Cardiol. 2007;30(3):135-40. Epub 2007/03/28. doi: 10.1002/clc.20058. PubMed PMID: 17385705; PMCID: PMC6652880. 17385705 PMC6652880

[108] Yeboah J, Folsom AR, Burke GL, Johnson C, Polak JF, Post W, Lima JA, Crouse JR, Herrington DM. Predictive value of brachial flow-mediated dilation for incident cardiovascular events in a population-based study: the multi-ethnic study of atherosclerosis. Circulation. 2009;120(6):502-9. Epub 2009/07/29. doi: 10.1161/CIRCULATIONAHA.109.864801. PubMed PMID: 19635967; PMCID: PMC2740975. 19635967 PMC2740975

[109] Wang X, Guo F, Li G, Cao Y, Fu H. Prognostic role of brachial reactivity in patients with ST myocardial infarction after percutaneous coronary intervention. Coron Artery Dis. 2009;20(7):467-72. Epub 2009/07/31. doi: 10.1097/MCA.0b013e32832e5c61. PubMed PMID: 19641459. 19641459

[110] Xu Y, Arora RC, Hiebert BM, Lerner B, Szwajcer A, McDonald K, Rigatto C, Komenda P, Sood MM, Tangri N. Non-invasive endothelial function testing and the risk of adverse outcomes: a systematic review and meta-analysis. Eur Heart J Cardiovasc Imaging. 2014;15(7):736-46. Epub 2014/01/09. doi: 10.1093/ehjci/jet256. PubMed PMID: 24399339. 24399339

[111] Vasilieva E, Vorobyeva I, Lebedeva A, Urazovskaya I, Kalinskaya A, Skrypnik D, Shpektor A. Brachial artery flow-mediated dilation in patients with Tako-tsubo cardiomyopathy. Am J Med. 2011;124(12):1176-9. Epub 2011/11/26. doi: 10.1016/j.amjmed.2011.05.033. PubMed PMID: 22114832. 22114832

[112] Amato M, Frigerio B, Castelnuovo S, Ravani A, Sansaro D, Tremoli E, Squellerio I, Cavalca V, Veglia F, Sirtori CR, Werba JP, Baldassarre D. Effects of smoking regular or light cigarettes on brachial artery flow-mediated dilation. Atherosclerosis. 2013;228(1):153-60. Epub 2013/03/27. doi: 10.1016/j.atherosclerosis.2013.02.037. PubMed PMID: 23528831. 23528831

[113] Weiner SD, Ahmed HN, Jin Z, Cushman M, Herrington DM, Nelson JC, Di Tullio MR, Homma S. Systemic inflammation and brachial artery endothelial function in the Multi-Ethnic Study of Atherosclerosis (MESA). Heart. 2014;100(11):862-6. Epub 2014/04/10. doi: 10.1136/heartjnl-2013-304893. PubMed PMID: 24714919. 24714919

[114] Altun I, Oz F, Arkaya SC, Altun I, Bilge AK, Umman B, Turkoglu UM. Effect of statins on endothelial function in patients with acute coronary syndrome: a prospective study using adhesion molecules and flow-mediated dilatation. J Clin Med Res. 2014;6(5):354-61. Epub 2014/08/12. doi: 10.14740/jocmr1863w. PubMed PMID: 25110539; PMCID: PMC4125330. 25110539 PMC4125330

[115] Gimbrone MA Jr., Garcia-Cardena G. Endothelial Cell Dysfunction and the Pathobiology of Atherosclerosis. Circ Res. 2016;118(4):620-36. Epub 2016/02/20. doi: 10.1161/CIRCRESAHA.115.306301. PubMed PMID: 26892962; PMCID: PMC4762052. 26892962 PMC4762052

[116] Jensen HA, Mehta JL. Endothelial cell dysfunction as a novel therapeutic target in atherosclerosis. Expert Rev Cardiovasc Ther. 2016;14(9):1021-33. Epub 2016/07/01. doi: 10.1080/14779072.2016.1207527. PubMed PMID: 27362558. 27362558

[117] Bentz GL, Jarquin-Pardo M, Chan G, Smith MS, Sinzger C, Yurochko AD. Human cytomegalovirus (HCMV) infection of endothelial cells promotes naive monocyte extravasation and transfer of productive virus to enhance hematogenous dissemination of HCMV. J Virol. 2006;80(23):11539-55. Epub 2006/09/22. doi: 10.1128/JVI.01016-06. PubMed PMID: 16987970; PMCID: PMC1642592. 16987970 PMC1642592

[118] DuRose JB, Li J, Chien S, Spector DH. Infection of vascular endothelial cells with human cytomegalovirus under fluid shear stress reveals preferential entry and spread of virus in flow conditions simulating atheroprone regions of the artery. J Virol. 2012;86(24):13745-55. Epub 2012/10/12. doi: 10.1128/JVI.02244-12. PubMed PMID: 23055562; PMCID: PMC3503096. 23055562 PMC3503096

[119] Lunardi C, Dolcino M, Peterlana D, Bason C, Navone R, Tamassia N, Tinazzi E, Beri R, Corrocher R, Puccetti A. Endothelial cells' activation and apoptosis induced by a subset of antibodies against human cytomegalovirus: relevance to the pathogenesis of atherosclerosis. PLoS One. 2007;2(5):e473. Epub 2007/05/31. doi: 10.1371/journal.pone.0000473. PubMed PMID: 17534423; PMCID: PMC1868596. 17534423 PMC1868596

[120] Tabata T, Kawakatsu H, Maidji E, Sakai T, Sakai K, Fang-Hoover J, Aiba M, Sheppard D, Pereira L. Induction of an epithelial integrin alphavbeta6 in human cytomegalovirus-infected endothelial cells leads to activation of transforming growth factor-beta1 and increased collagen production. Am J Pathol. 2008;172(4):1127-40. Epub 2008/03/20. doi: 10.2353/ajpath.2008.070448. PubMed PMID: 18349127; PMCID: PMC2276431. 18349127 PMC2276431

[121] Ghielmetti M, Millard AL, Haeberli L, Bossart W, Seebach JD, Schneider MK, Mueller NJ. Human CMV infection of porcine endothelial cells increases adhesion receptor expression and human leukocyte recruitment. Transplantation. 2009;87(12):1792-800. Epub 2009/06/23. doi: 10.1097/TP.0b013e3181a75a41. PubMed PMID: 19543055. 19543055

[122] Popovic M, Smiljanic K, Dobutovic B, Syrovets T, Simmet T, Isenovic ER. Human cytomegalovirus infection and atherothrombosis. J Thromb Thrombolysis. 2012;33(2):160-72. Epub 2011/12/14. doi: 10.1007/s11239-011-0662-x. PubMed PMID: 22161772. 22161772

[123] Gustafsson KLR, Renne T, Soderberg-Naucler C, Butler LM. Human cytomegalo-virus replication induces endothelial cell interleukin-11. Cytokine. 2018;111:563-6. Epub 2018/05/29. doi: 10.1016/j.cyto.2018.05.018. PubMed PMID: 29807687; PMCID: PMC6299253. 29807687 PMC6299253

[124] Bolovan-Fritts CA, Trout RN, Spector SA. High T-cell response to human cytomegalovirus induces chemokine-mediated endothelial cell damage. Blood. 2007;110(6):1857-63. Epub 2007/05/24. doi: 10.1182/blood-2007-03-078881. PubMed PMID: 17519388; PMCID: PMC1976357. 17519388 PMC1976357

[125] Khoretonenko MV, Leskov IL, Jennings SR, Yurochko AD, Stokes KY. Cytomegalo-virus infection leads to microvascular dysfunction and exacerbates hypercholesterolemia-induced responses. Am J Pathol. 2010;177(4):2134-44. Epub 2010/08/31. doi: 10.2353/ajpath.2010.100307. PubMed PMID: 20802174; PMCID: PMC2947306. 20802174 PMC2947306

[126] Gombos RB, Brown JC, Teefy J, Gibeault RL, Conn KL, Schang LM, Hemmings DG. Vascular dysfunction in young, mid-aged and aged mice with latent cytomegalovirus infections. Am J Physiol Heart Circ Physiol. 2013;304(2):H183-94. Epub 2012/11/06. doi: 10.1152/ajpheart.00461.2012. PubMed PMID: 23125213; PMCID: PMC3543669. 23125213 PMC3543669

[127] Straat K, de Klark R, Gredmark-Russ S, Eriksson P, Soderberg-Naucler C. Infection with human cytomegalovirus alters the MMP-9/TIMP-1 balance in human macrophages. J Virol. 2009;83(2):830-5. Epub 2008/10/24. doi: 10.1128/JVI.01363-08. PubMed PMID: 18945772; PMCID: PMC2612361. 18945772 PMC2612361

[128] Rahbar A, Soderberg-Naucler C. Human cytomegalovirus infection of endothelial cells triggers platelet adhesion and aggregation. J Virol. 2005;79(4):2211-20. Epub 2005/02/01. doi: 10.1128/JVI.79.4.2211-2220.2005. PubMed PMID: 15681423; PMCID: PMC546536. 15681423 PMC546536

[129] Alfawaz A. Cytomegalovirus-related corneal endotheliitis: A review article. Saudi J Ophthalmol. 2013;27(1):47-9. Epub 2013/08/22. doi: 10.1016/j.sjopt.2011.10.001. PubMed PMID: 23964187; PMCID: PMC3729553. 23964187 PMC3729553

[130] Grahame-Clarke C, Chan NN, Andrew D, Ridgway GL, Betteridge DJ, Emery V, Colhoun HM, Vallance P. Human cytomegalovirus seropositivity is associated with impaired vascular function. Circulation. 2003;108(6):678-83. Epub 2003/08/06. doi: 10.1161/01.CIR.0000084505.54603.C7. PubMed PMID: 12900349. 12900349

[131] Haarala A, Kahonen M, Lehtimaki T, Aittoniemi J, Jylhava J, Hutri-Kahonen N, Taittonen L, Laitinen T, Juonala M, Viikari J, Raitakari OT, Hurme M. Relation of high cytomegalovirus antibody titres to blood pressure and brachial artery flow-mediated dilation in young men: the Cardiovascular Risk in Young Finns Study. Clin Exp Immunol. 2012;167(2):309-16. Epub 2012/01/13. doi: 10.1111/j.1365-2249.2011.04513.x. PubMed PMID: 22236008; PMCID: PMC3278698. 22236008 PMC3278698

[132] Khairy P, Rinfret S, Tardif JC, Marchand R, Shapiro S, Brophy J, Dupuis J. Absence of association between infectious agents and endothelial function in healthy young men. Circulation. 2003;107(15):1966-71. Epub 2003/04/19. doi: 10.1161/01.CIR.0000064895.89033.97. PubMed PMID: 12681997. 12681997

[133] Oshima T, Ozono R, Yano Y, Oishi Y, Teragawa H, Higashi Y, Yoshizumi M, Kambe M. Association of Helicobacter pylori infection with systemic inflammation and endothelial dysfunction in healthy male subjects. J Am Coll Cardiol. 2005;45(8):1219-22. Epub 2005/04/20. doi: 10.1016/j.jacc.2005.01.019. PubMed PMID: 15837252. 15837252

[134] Simmonds J, Fenton M, Dewar C, Ellins E, Storry C, Cubitt D, Deanfield J, Klein N, Halcox J, Burch M. Endothelial dysfunction and cytomegalovirus replication in pediatric heart transplantation. Circulation. 2008;117(20):2657-61. Epub 2008/05/14. doi: 10.1161/CIRCULATIONAHA.107.718874. PubMed PMID: 18474812. 18474812

[135] Petrakopoulou P, Kubrich M, Pehlivanli S, Meiser B, Reichart B, von Scheidt W, Weis M. Cytomegalovirus infection in heart transplant recipients is associated with impaired endothelial function. Circulation. 2004;110(11 Suppl 1):II207-12. Epub 2004/09/15. doi: 10.1161/01.CIR.0000138393.99310.1c. PubMed PMID: 15364864. 15364864

[136] Weis M, Kledal TN, Lin KY, Panchal SN, Gao SZ, Valantine HA, Mocarski ES, Cooke JP. Cytomegalovirus infection impairs the nitric oxide synthase pathway: role of asymmetric dimethylarginine in transplant arteriosclerosis. Circulation. 2004;109(4):500-5. Epub 2004/01/21. doi: 10.1161/01.CIR.0000109692.16004.AF. PubMed PMID: 14732750. 14732750

[137] Prasad A, Zhu J, Halcox JP, Waclawiw MA, Epstein SE, Quyyumi AA. Predisposition to atherosclerosis by infections: role of endothelial dysfunction. Circulation. 2002;106(2):184-90. Epub 2002/07/10. doi: 10.1161/01.cir.0000021125.83697.21. PubMed PMID: 12105156. 12105156

[138] Nikitskaya E, Lebedeva A, Ivanova O, Maryukhnich E, Shpektor A, Grivel JC, Margolis L, Vasilieva E. Cytomegalovirus-Productive Infection Is Associated With Acute Coronary Syndrome. J Am Heart Assoc. 2016;5(8). Epub 2016/08/21. doi: 10.1161/JAHA.116.003759. PubMed PMID: 27543799; PMCID: PMC5015295. PMC5015295 27543799

[139] Lebedeva A, Maryukhnich E, Grivel JC, Vasilieva E, Margolis L, Shpektor A. Productive Cytomegalovirus Infection Is Associated With Impaired Endothelial Function in ST-Elevation Myocardial Infarction. Am J Med. 2020;133(1):133-42. Epub 2019/07/12. doi: 10.1016/j.amjmed.2019.06.021. PubMed PMID: 31295440; PMCID: PMC6940528. 31295440 PMC6940528

[140] Younes SA, Freeman ML, Mudd JC, Shive CL, Reynaldi A, Panigrahi S, Estes JD, Deleage C, Lucero C, Anderson J, Schacker TW, Davenport MP, McCune JM, Hunt PW, Lee SA, Serrano-Villar S, Debernardo RL, Jacobson JM, Canaday DH, Sekaly RP, Rodriguez B, Sieg SF, Lederman MM. IL-15 promotes activation and expansion of CD8+ T cells in HIV-1 infection. J Clin Invest. 2016;126(7):2745-56. Epub 2016/06/21. doi: 10.1172/JCI85996. PubMed PMID: 27322062; PMCID: PMC4922693. 27322062 PMC4922693

[141] Pangrazzi L, Naismith E, Meryk A, Keller M, Jenewein B, Trieb K, Grubeck-Loebenstein B. Increased IL-15 Production and Accumulation of Highly Differentiated CD8(+) Effector/Memory T Cells in the Bone Marrow of Persons with Cytomegalovirus. Front Immunol. 2017;8:715. Epub 2017/07/05. doi: 10.3389/fimmu.2017.00715. PubMed PMID: 28674537; PMCID: PMC5474847. 28674537 PMC5474847

[142] Baumann NS, Torti N, Welten SPM, Barnstorf I, Borsa M, Pallmer K, Oduro JD, Cicin-Sain L, Ikuta K, Ludewig B, Oxenius A. Tissue maintenance of CMV-specific inflationary memory T cells by IL-15. PLoS Pathog. 2018;14(4):e1006993. Epub 2018/04/14. doi: 10.1371/journal.ppat.1006993. PubMed PMID: 29652930; PMCID: PMC5919076. 29652930 PMC5919076

[143] Kaibe M, Ohishi M, Ito N, Yuan M, Takagi T, Terai M, Tatara Y, Komai N, Rakugi H, Ogihara T. Serum interleukin-15 concentration in patients with essential hypertension. Am J Hypertens. 2005;18(8):1019-25. Epub 2005/08/20. doi: 10.1016/j.amjhyper.2005.02.014. PubMed PMID: 16109314. 16109314

[144] Dozio E, Malavazos AE, Vianello E, Briganti S, Dogliotti G, Bandera F, Giacomazzi F, Castelvecchio S, Menicanti L, Sigruener A, Schmitz G, Corsi Romanelli MM. Interleukin-15 and soluble interleukin-15 receptor alpha in coronary artery disease patients: association with epicardial fat and indices of adipose tissue distribution. PLoS One. 2014;9(3):e90960. Epub 2014/03/08. doi: 10.1371/journal.pone.0090960. PubMed PMID: 24603895; PMCID: PMC3948349. 24603895 PMC3948349

[145] van Es T, van Puijvelde GH, Michon IN, van Wanrooij EJ, de Vos P, Peterse N, van Berkel TJ, Kuiper J. IL-15 aggravates atherosclerotic lesion development in LDL receptor deficient mice. Vaccine. 2011;29(5):976-83. Epub 2010/12/01. doi: 10.1016/j.vaccine.2010.11.037. PubMed PMID: 21115056. 21115056

[146] Houtkamp MA, van Der Wal AC, de Boer OJ, van Der Loos CM, de Boer PA, Moorman AF, Becker AE. Interleukin-15 expression in atherosclerotic plaques: an alternative pathway for T-cell activation in atherosclerosis? Arteriosclerosis, thrombosis, and vascular biology. 2001;21(7):1208-13. Epub 2001/07/14. doi: 10.1161/hq0701.092162. PubMed PMID: 11451753. 11451753

[147] Wuttge DM, Eriksson P, Sirsjo A, Hansson GK, Stemme S. Expression of interleukin-15 in mouse and human atherosclerotic lesions. Am J Pathol. 2001;159(2):417-23. Epub 2001/08/04. doi: 10.1016/S0002-9440(10)61712-9. PubMed PMID: 11485899; PMCID: PMC1850554. 11485899 PMC1850554

[148] Sieg SF, Bazdar DA, Zidar D, Freeman M, Lederman MM, Funderburg NT. Highly oxidized low-density lipoprotein mediates activation of monocytes but does not confer interleukin-1beta secretion nor interleukin-15 transpresentation function. Immunology. 2020;159(2):221-30. Epub 2019/10/31. doi: 10.1111/imm.13142. PubMed PMID: 31663113; PMCID: PMC6954695. 31663113 PMC6954695

[149] Walker JD, Maier CL, Pober JS. Cytomegalovirus-infected human endothelial cells can stimulate allogeneic CD4+ memory T cells by releasing antigenic exosomes. Journal of immunology. 2009;182(3):1548-59. Epub 2009/01/22. doi: 10.4049/jimmunol.182.3.1548. PubMed PMID: 19155503; PMCID: PMC2630120. PMC2630120 19155503

[150] Zicari S, Arakelyan A, Palomino RAN, Fitzgerald W, Vanpouille C, Lebedeva A, Schmitt A, Bomsel M, Britt W, Margolis L. Human cytomegalovirus-infected cells release extracellular vesicles that carry viral surface proteins. Virology. 2018;524:97-105. Epub 2018/08/31. doi: 10.1016/j.virol.2018.08.008. PubMed PMID: 30165311; PMCID: PMC6258833. 30165311 PMC6258833

[151] Vagida M, Arakelyan A, Lebedeva A, Grivel JC, Shpektor A, Vasilieva E, Margolis L. Flow analysis of individual blood extracellular vesicles in acute coronary syndrome. Platelets. 2017;28(2):165-73. Epub 2016/10/19. doi: 10.1080/09537104.2016.1212002. PubMed PMID: 27595614; PMCID: PMC5811196. 27595614 PMC5811196

[152] Dargan DJ, Subak-Sharpe JH. The effect of herpes simplex virus type 1 L-particles on virus entry, replication, and the infectivity of naked herpesvirus DNA. Virology. 1997;239(2):378-88. Epub 1998/01/22. doi: 10.1006/viro.1997.8893. PubMed PMID: 9434728. 9434728

[153] Rozmyslowicz T, Majka M, Kijowski J, Murphy SL, Conover DO, Poncz M, Ratajczak J, Gaulton GN, Ratajczak MZ. Platelet- and megakaryocyte-derived microparticles transfer CXCR4 receptor to CXCR4-null cells and make them susceptible to infection by X4-HIV. Aids. 2003;17(1):33-42. Epub 2002/12/13. doi: 10.1097/00002030-200301030-00006. PubMed PMID: 12478067. 12478067

[154] Wiley RD, Gummuluru S. Immature dendritic cell-derived exosomes can mediate HIV-1 trans infection. Proc Natl Acad Sci U S A. 2006;103(3):738-43. Epub 2006/01/13. doi: 10.1073/pnas.0507995103. PubMed PMID: 16407131; PMCID: PMC1334656. 16407131 PMC1334656

[155] Ramakrishnaiah V, Thumann C, Fofana I, Habersetzer F, Pan Q, de Ruiter PE, Willemsen R, Demmers JA, Stalin Raj V, Jenster G, Kwekkeboom J, Tilanus HW, Haagmans BL, Baumert TF, van der Laan LJ. Exosome-mediated transmission of hepatitis C virus between human hepatoma Huh7.5 cells. Proc Natl Acad Sci U S A. 2013;110(32):13109-13. Epub 2013/07/24. doi: 10.1073/pnas.1221899110. PubMed PMID: 23878230; PMCID: PMC3740869. 23878230 PMC3740869

[156] Meckes DG Jr., Raab-Traub N. Microvesicles and viral infection. J Virol. 2011;85(24):12844-54. Epub 2011/10/07. doi: 10.1128/JVI.05853-11. PubMed PMID: 21976651; PMCID: PMC3233125. 21976651 PMC3233125

[157] Suades R, Padro T, Alonso R, Mata P, Badimon L. Lipid-lowering therapy with statins reduces microparticle shedding from endothelium, platelets and inflammatory cells. Thromb Haemost. 2013;110(2):366-77. Epub 2013/06/07. doi: 10.1160/TH13-03-0238. PubMed PMID: 23740299. 23740299

[158] Ponroy N, Taveira A, Mueller NJ, Millard AL. Statins demonstrate a broad anti-cytomegalovirus activity in vitro in ganciclovir-susceptible and resistant strains. J Med Virol. 2015;87(1):141-53. Epub 2014/07/01. doi: 10.1002/jmv.23998. PubMed PMID: 24976258. 24976258

[159] Yi L, Wang JW, Zhao RG, Tuo HZ, Feng ZJ, Wang DX. [Fluvastatin's effect on athero-genesis in apolipoprotein-E knockout mice infected by cytomegalovirus]. Zhonghua Shi Yan He Lin Chuang Bing Du Xue Za Zhi. 2010;24(6):433-5. Epub 2011/05/25. PubMed PMID: 21604568. 21604568

[160] Kloppenburg G, de Graaf R, Herngreen S, Grauls G, Bruggeman C, Stassen F. Cytomegalovirus aggravates intimal hyperplasia in rats by stimulating smooth muscle cell proliferation. Microbes Infect. 2005;7(2):164-70. Epub 2005/02/18. doi: 10.1016/j.micinf.2004.10.008. PubMed PMID: 15716015. 15716015

[161] Lemstrom KB, Bruning JH, Bruggeman CA, Lautenschlager IT, Hayry PJ. Cytomegalovirus infection enhances smooth muscle cell proliferation and intimal thickening of rat aortic allografts. J Clin Invest. 1993;92(2):549-58. Epub 1993/08/01. doi: 10.1172/JCI116622. PubMed PMID: 8394384; PMCID: PMC294886. 8394384 PMC294886

[162] Lemstrom KB, Koskinen PK, Bruning JH, Bruggeman CA, Lautenschlager IT, Hayry PJ. Effect of ganciclovir prophylaxis on cytomegalovirus-enhanced allograft arteriosclerosis. Transpl Int. 1994;7 Suppl 1:S383-4. Epub 1994/01/01. doi: 10.1111/j.1432-2277.1994.tb01398.x. PubMed PMID: 11271259. 11271259

[163] Lemstrom K, Koskinen P, Krogerus L, Daemen M, Bruggeman C, Hayry P. Cytomegalovirus antigen expression, endothelial cell proliferation, and intimal thickening in rat cardiac allografts after cytomegalovirus infection. Circulation. 1995;92(9):2594-604. Epub 1995/11/01. doi: 10.1161/01.cir.92.9.2594. PubMed PMID: 7586362. 7586362

[164] Tang-Feldman YJ, Lochhead SR, Lochhead GR, Yu C, George M, Villablanca AC, Pomeroy C. Murine cytomegalovirus (MCMV) infection upregulates P38 MAP kinase in aortas of Apo E KO mice: a molecular mechanism for MCMV-induced acceleration of atherosclerosis. J Cardiovasc Transl Res. 2013;6(1):54-64. Epub 2012/11/30. doi: 10.1007/s12265-012-9428-x. PubMed PMID: 23192592; PMCID: PMC4591060. 23192592 PMC4591060

[165] Springer KL, Weinberg A. Cytomegalovirus infection in the era of HAART: fewer reactivations and more immunity. J Antimicrob Chemother. 2004;54(3):582-6. Epub 2004/07/30. doi: 10.1093/jac/dkh396. PubMed PMID: 15282241. 15282241

[166] Lederman MM, Funderburg NT, Sekaly RP, Klatt NR, Hunt PW. Residual immune dysregulation syndrome in treated HIV infection. Advances in immunology. 2013;119:51-83. Epub 2013/07/28. doi: 10.1016/B978-0-12-407707-2.00002-3. PubMed PMID: 23886064; PMCID: PMC4126613. 23886064 PMC4126613

[167] Barrett L, Stapleton SN, Fudge NJ, Grant MD. Immune resilience in HIV-infected individuals seronegative for cytomegalovirus. Aids. 2014;28(14):2045-9. Epub 2014/09/30. doi: 10.1097/QAD.0000000000000405. PubMed PMID: 25265072. 25265072

[168] Hunt PW, Martin JN, Sinclair E, Epling L, Teague J, Jacobson MA, Tracy RP, Corey L, Deeks SG. Valganciclovir reduces T cell activation in HIV-infected individuals with incomplete CD4+ T cell recovery on antiretroviral therapy. J Infect Dis. 2011;203(10):1474-83. Epub 2011/04/20. doi: 10.1093/infdis/jir060. PubMed PMID: 21502083; PMCID: PMC3080892. 21502083 PMC3080892

[169] Maidji E, Somsouk M, Rivera JM, Hunt PW, Stoddart CA. Replication of CMV in the gut of HIV-infected individuals and epithelial barrier dysfunction. PLoS Pathog. 2017;13(2):e1006202. Epub 2017/02/28. doi: 10.1371/journal.ppat.1006202. PubMed PMID: 28241080; PMCID: PMC5328284. 28241080 PMC5328284

[170] Marty FM, Ljungman P, Chemaly RF, Maertens J, Dadwal SS, Duarte RF, Haider S, Ullmann AJ, Katayama Y, Brown J, Mullane KM, Boeckh M, Blumberg EA, Einsele H, Snydman DR, Kanda Y, DiNubile MJ, Teal VL, Wan H, Murata Y, Kartsonis NA, Leavitt RY, Badshah C. Letermovir Prophylaxis for Cytomegalovirus in Hematopoietic-Cell Transplantation. N Engl J Med. 2017;377(25):2433-44. Epub 2017/12/07. doi: 10.1056/NEJMoa1706640. PubMed PMID: 29211658. 29211658

[171] Bowman LJ, Melaragno JI, Brennan DC. Letermovir for the management of cytomegalovirus infection. Expert Opin Investig Drugs. 2017;26(2):235-41. Epub 2016/12/22. doi: 10.1080/13543784.2017.1274733. PubMed PMID: 27998189. 27998189

[172] Hutter G, Nowak D, Mossner M, Ganepola S, Mussig A, Allers K, Schneider T, Hofmann J, Kucherer C, Blau O, Blau IW, Hofmann WK, Thiel E. Long-term control of HIV by CCR5 Delta32/Delta32 stem-cell transplantation. N Engl J Med. 2009;360(7):692-8. Epub 2009/02/14. doi: 10.1056/NEJMoa0802905. PubMed PMID: 19213682. 19213682

[173] Gupta RK, Abdul-Jawad S, McCoy LE, Mok HP, Peppa D, Salgado M, Martinez-Picado J, Nijhuis M, Wensing AMJ, Lee H, Grant P, Nastouli E, Lambert J, Pace M, Salasc F, Monit C, Innes AJ, Muir L, Waters L, Frater J, Lever AML, Edwards SG, Gabriel IH, Olavarria E. HIV-1 remission following CCR5Delta32/Delta32 haematopoietic stem-cell transplantation. Nature. 2019;568(7751):244-8. Epub 2019/03/06. doi: 10.1038/s41586-019-1027-4. PubMed PMID: 30836379; PMCID: PMC7275870. 30836379 PMC7275870

[174] Seif M, Einsele H, Loffler J. CAR T Cells Beyond Cancer: Hope for Immunomodulatory Therapy of Infectious Diseases. Front Immunol. 2019;10:2711. Epub 2019/12/12. doi: 10.3389/fimmu.2019.02711. PubMed PMID: 31824500; PMCID: PMC6881243. 31824500 PMC6881243

[175] Rohaan MW, Wilgenhof S, Haanen J. Adoptive cellular therapies: the current landscape. Virchows Arch. 2019;474(4):449-61. Epub 2018/11/25. doi: 10.1007/s00428-018-2484-0. PubMed PMID: 30470934; PMCID: PMC6447513. 30470934 PMC6447513

[176] Full F, Lehner M, Thonn V, Goetz G, Scholz B, Kaufmann KB, Mach M, Abken H, Holter W, Ensser A. T cells engineered with a cytomegalovirus-specific chimeric immunoreceptor. J Virol. 2010;84(8):4083-8. Epub 2010/02/12. doi: 10.1128/JVI.02117-09. PubMed PMID: 20147393; PMCID: PMC2849495. 20147393 PMC2849495

[177] Proff J, Walterskirchen C, Brey C, Geyeregger R, Full F, Ensser A, Lehner M, Holter W. Cytomegalovirus-Infected Cells Resist T Cell Mediated Killing in an HLA-Recognition Independent Manner. Front Microbiol. 2016;7:844. Epub 2016/07/05. doi: 10.3389/fmicb.2016.00844. PubMed PMID: 27375569; PMCID: PMC4899442. 27375569 PMC4899442

[178] Proff J, Brey CU, Ensser A, Holter W, Lehner M. Turning the tables on cytomegalovirus: targeting viral Fc receptors by CARs containing mutated CH2-CH3 IgG spacer domains. J Transl Med. 2018;16(1):26. Epub 2018/02/10. doi: 10.1186/s12967-018-1394-x. PubMed PMID: 29422056; PMCID: PMC5804023. 29422056 PMC5804023

[179] Olbrich H, Theobald SJ, Slabik C, Gerasch L, Schneider A, Mach M, Shum T, Mamonkin M, Stripecke R. Adult and Cord Blood-Derived High-Affinity gB-CAR-T Cells Effectively React Against Human Cytomegalovirus Infections. Hum Gene Ther. 2020;31(7-8):423-39. Epub 2020/03/12. doi: 10.1089/hum.2019.149. PubMed PMID: 32159399; PMCID: PMC7194322. 32159399 PMC7194322

[180] Alvi RM, Frigault MJ, Fradley MG, Jain MD, Mahmood SS, Awadalla M, Lee DH, Zlotoff DA, Zhang L, Drobni ZD, Hassan MZO, Bassily E, Rhea I, Ismail-Khan R, Mulligan CP, Banerji D, Lazaryan A, Shah BD, Rokicki A, Raje N, Chavez JC, Abramson J, Locke FL, Neilan TG. Cardiovascular Events Among Adults Treated With Chimeric Antigen Receptor T-Cells (CAR-T). J Am Coll Cardiol. 2019;74(25):3099-108. Epub 2019/12/21. doi: 10.1016/j.jacc.2019.10.038. PubMed PMID: 31856966; PMCID: PMC6938409. 31856966 PMC6938409

[181] Jamal FA, Khaled SK. The Cardiovascular Complications of Chimeric Antigen Receptor T Cell Therapy. Curr Hematol Malig Rep. 2020;15(2):130-2. Epub 2020/02/06. doi: 10.1007/s11899-020-00567-4. PubMed PMID: 32016789. 32016789

[182] Collin M. Immune checkpoint inhibitors: a patent review (2010-2015). Expert Opin Ther Pat. 2016;26(5):555-64. Epub 2016/04/08. doi: 10.1080/13543776.2016.1176150. PubMed PMID: 27054314. 27054314

[183] Fritz JM, Lenardo MJ. Development of immune checkpoint therapy for cancer. The Journal of experimental medicine. 2019;216(6):1244-54. Epub 2019/05/10. doi: 10.1084/jem.20182395. PubMed PMID: 31068379; PMCID: PMC6547853. 31068379 PMC6547853

[184] Buggert M, Tauriainen J, Yamamoto T, Frederiksen J, Ivarsson MA, Michaelsson J, Lund O, Hejdeman B, Jansson M, Sonnerborg A, Koup RA, Betts MR, Karlsson AC. T-bet and Eomes are differentially linked to the exhausted phenotype of CD8+ T cells in HIV infection. PLoS Pathog. 2014;10(7):e1004251. Epub 2014/07/18. doi: 10.1371/journal.ppat.1004251. PubMed PMID: 25032686; PMCID: PMC4102564. 25032686 PMC4102564

[185] Postow MA, Sidlow R, Hellmann MD. Immune-Related Adverse Events Associated with Immune Checkpoint Blockade. N Engl J Med. 2018;378(2):158-68. Epub 2018/01/11. doi: 10.1056/NEJMra1703481. PubMed PMID: 29320654. 29320654

[186] Cosman D, Fanger N, Borges L, Kubin M, Chin W, Peterson L, Hsu ML. A novel immunoglobulin superfamily receptor for cellular and viral MHC class I molecules. Immunity. 1997;7(2):273-82. Epub 1997/08/01. doi: 10.1016/s1074-7613(00)80529-4. PubMed PMID: 9285411. 9285411

[187] Ince MN, Harnisch B, Xu Z, Lee SK, Lange C, Moretta L, Lederman M, Lieberman J. Increased expression of the natural killer cell inhibitory receptor CD85j/ILT2 on antigen-specific effector CD8 T cells and its impact on CD8 T-cell function. Immunology. 2004;112(4):531-42. Epub 2004/07/24. doi: 10.1046/j.1365-2567.2004.01907.x. PubMed PMID: 15270723; PMCID: PMC1782522. 15270723 PMC1782522

[188] Chapman TL, Heikeman AP, Bjorkman PJ. The inhibitory receptor LIR-1 uses a common binding interaction to recognize class I MHC molecules and the viral homolog UL18. Immunity. 1999;11(5):603-13. Epub 1999/12/11. doi: 10.1016/s1074-7613(00)80135-1. PubMed PMID: 10591185. 10591185

[189] Wagner CS, Ljunggren HG, Achour A. Immune modulation by the human cytomegalovirus-encoded molecule UL18, a mystery yet to be solved. Journal of immunology. 2008;180(1):19-24. Epub 2007/12/22. doi: 10.4049/jimmunol.180.1.19. PubMed PMID: 18096997. 18096997

[190] Schleiss MR, Permar SR, Plotkin SA. Progress toward Development of a Vaccine against Congenital Cytomegalovirus Infection. Clin Vaccine Immunol. 2017;24(12). Epub 2017/10/20. doi: 10.1128/CVI.00268-17. PubMed PMID: 29046308; PMCID: PMC5717185. PMC5717185 29046308

[191] Plotkin SA, Boppana SB. Vaccination against the human cytomegalovirus. Vaccine. 2019;37(50):7437-42. Epub 2018/04/07. doi: 10.1016/j.vaccine.2018.02.089. PubMed PMID: 29622379; PMCID: PMC6892274. 29622379 PMC6892274

[192] La Rosa C, Longmate J, Martinez J, Zhou Q, Kaltcheva TI, Tsai W, Drake J, Carroll M, Wussow F, Chiuppesi F, Hardwick N, Dadwal S, Aldoss I, Nakamura R, Zaia JA, Diamond DJ. MVA vaccine encoding CMV antigens safely induces durable expansion of CMV-specific T cells in healthy adults. Blood. 2017;129(1):114-25. Epub 2016/10/21. doi: 10.1182/blood-2016-07-729756. PubMed PMID: 27760761; PMCID: PMC5216266. 27760761 PMC5216266

[193] Aldoss I, La Rosa C, Baden LR, Longmate J, Ariza-Heredia EJ, Rida WN, Lingaraju CR, Zhou Q, Martinez J, Kaltcheva T, Dagis A, Hardwick N, Issa NC, Farol L, Nademanee A, Al Malki MM, Forman S, Nakamura R, Diamond DJ, Group TVS. Poxvirus Vectored Cytomegalovirus Vaccine to Prevent Cytomegalovirus Viremia in Transplant Recipients: A Phase 2, Randomized Clinical Trial. Ann Intern Med. 2020;172(5):306-16. Epub 2020/02/11. doi: 10.7326/M19-2511. PubMed PMID: 32040960. 32040960 PMC9074089

[194] Melnick JL, Hu C, Burek J, Adam E, DeBakey ME. Cytomegalovirus DNA in arterial walls of patients with atherosclerosis. J Med Virol. 1994;42(2):170-4. Epub 1994/02/01. doi: 10.1002/jmv.1890420213. PubMed PMID: 8158112. 8158112

[195] Xenaki E, Hassoulas J, Apostolakis S, Sourvinos G, Spandidos DA. Detection of cytomegalovirus in atherosclerotic plaques and nonatherosclerotic arteries. Angiology. 2009;60(4):504-8. Epub 2008/09/27. doi: 10.1177/0003319708322390. PubMed PMID: 18818234. 18818234

[196] Collaborators GBDCoD. Global, regional, and national age-sex-specific mortality for 282 causes of death in 195 countries and territories, 1980-2017: a systematic analysis for the Global Burden of Disease Study 2017. Lancet. 2018;392(10159):1736-88. Epub 2018/11/30. doi: 10.1016/S0140-6736(18)32203-7. PubMed PMID: 30496103; PMCID: PMC6227606. 30496103 PMC6227606

[197] Heidenreich PA, Trogdon JG, Khavjou OA, Butler J, Dracup K, Ezekowitz MD, Finkelstein EA, Hong Y, Johnston SC, Khera A, Lloyd-Jones DM, Nelson SA, Nichol G, Orenstein D, Wilson PW, Woo YJ, American Heart Association Advocacy Coordinating C, Stroke C, Council on Cardiovascular R, Intervention, Council on Clinical C, Council on E, Prevention, Council on A, Thrombosis, Vascular B, Council on C, Critical C, Perioperative, Resuscitation, Council on Cardiovascular N, Council on the Kidney in Cardiovascular D, Council on Cardiovascular S, Anesthesia, Interdisciplinary Council on Quality of C, Outcomes R. Forecasting the future of cardiovascular disease in the United States: a policy statement from the American Heart Association. Circulation. 2011;123(8):933-44. Epub 2011/01/26. doi: 10.1161/CIR.0b013e31820a55f5. PubMed PMID: 21262990. 21262990

[198] Ynga-Durand MA, Dekhtiarenko I, Cicin-Sain L. Vaccine Vectors Harnessing the Power of Cytomegaloviruses. Vaccines (Basel). 2019;7(4). Epub 2019/10/20. doi: 10.3390/vaccines7040152. PubMed PMID: 31627457; PMCID: PMC6963789. PMC6963789 31627457

[199] Caposio P, van den Worm S, Crawford L, Perez W, Kreklywich C, Gilbride RM, Hughes CM, Ventura AB, Ratts R, Marshall EE, Malouli D, Axthelm MK, Streblow D, Nelson JA, Picker LJ, Hansen SG, Fruh K. Characterization of a live-attenuated HCMV-based vaccine platform. Sci Rep. 2019;9(1):19236. Epub 2019/12/19. doi: 10.1038/s41598-019-55508-w. PubMed PMID: 31848362; PMCID: PMC6917771. 31848362 PMC6917771

[200] Hansen SG, Ford JC, Lewis MS, Ventura AB, Hughes CM, Coyne-Johnson L, Whiz-in N, Oswald K, Shoemaker R, Swanson T, Legasse AW, Chiuchiolo MJ, Parks CL, Axthelm MK, Nelson JA, Jarvis MA, Piatak M Jr., Lifson JD, Picker LJ. Profound early control of highly pathogenic SIV by an effector memory T-cell vaccine. Nature. 2011;473(7348):523-7. Epub 2011/05/13. doi: 10.1038/nature10003. PubMed PMID: 21562493; PMCID: PMC3102768. PMC3102768 21562493

[201] Klyushnenkova EN, Kouiavskaia DV, Parkins CJ, Caposio P, Botto S, Alexander RB, Jarvis MA. A cytomegalovirus-based vaccine expressing a single tumor-specific CD8+ T-cell epitope delays tumor growth in a murine model of prostate cancer. J Immunother. 2012;35(5):390-9. Epub 2012/05/12. doi: 10.1097/CJI.0b013e3182585d50. PubMed PMID: 22576344; PMCID: PMC3366429. 22576344 PMC3366429

[202] Hansen SG, Piatak M Jr., Ventura AB, Hughes CM, Gilbride RM, Ford JC, Oswald K, Shoemaker R, Li Y, Lewis MS, Gilliam AN, Xu G, Whizin N, Burwitz BJ, Planer SL, Turner JM, Legasse AW, Axthelm MK, Nelson JA, Fruh K, Sacha JB, Estes JD, Keele BF, Edlefsen PT, Lifson JD, Picker LJ. Immune clearance of highly pathogenic SIV infection. Nature. 2013;502(7469):100-4. Epub 2013/09/13. doi: 10.1038/nature12519. PubMed PMID: 24025770; PMCID: PMC3849456. 24025770 PMC3849456

[203] Qiu Z, Huang H, Grenier JM, Perez OA, Smilowitz HM, Adler B, Khanna KM. Cytomegalovirus-Based Vaccine Expressing a Modified Tumor Antigen Induces Potent Tumor-Specific CD8(+) T-cell Response and Protects Mice from Melanoma. Cancer Immunol Res. 2015;3(5):536-46. Epub 2015/01/31. doi: 10.1158/2326-6066.CIR-14-0044. PubMed PMID: 25633711. 25633711

[204] Grenier JM, Yeung ST, Qiu Z, Jellison ER, Khanna KM. Combining Adoptive Cell Therapy with Cytomegalovirus-Based Vaccine Is Protective against Solid Skin Tumors. Front Immunol. 2017;8:1993. Epub 2018/02/02. doi: 10.3389/fimmu.2017.01993. PubMed PMID: 29387061; PMCID: PMC5775971. 29387061 PMC5775971

[205] Hansen SG, Zak DE, Xu G, Ford JC, Marshall EE, Malouli D, Gilbride RM, Hughes CM, Ventura AB, Ainslie E, Randall KT, Selseth AN, Rundstrom P, Herlache L, Lewis MS, Park H, Planer SL, Turner JM, Fischer M, Armstrong C, Zweig RC, Valvo J, Braun JM, Shankar S, Lu L, Sylwester AW, Legasse AW, Messerle M, Jarvis MA, Amon LM, Aderem A, Alter G, Laddy DJ, Stone M, Bonavia A, Evans TG, Axthelm MK, Fruh K, Edlefsen PT, Picker LJ. Prevention of tuberculosis in rhesus macaques by a cytomegalovirus-based vaccine. Nat Med. 2018;24(2):130-43. Epub 2018/01/16. doi: 10.1038/nm.4473. PubMed PMID: 29334373; PMCID: PMC5909823. 29334373 PMC5909823

[206] Hansen SG, Marshall EE, Malouli D, Ventura AB, Hughes CM, Ainslie E, Ford JC, Morrow D, Gilbride RM, Bae JY, Legasse AW, Oswald K, Shoemaker R, Berkemeier B, Bosche WJ, Hull M, Womack J, Shao J, Edlefsen PT, Reed JS, Burwitz BJ, Sacha JB, Axthelm MK, Fruh K, Lifson JD, Picker LJ. A live-attenuated RhCMV/SIV vaccine shows long-term efficacy against heterologous SIV challenge. Science translational medicine. 2019;11(501). Epub 2019/07/19. doi: 10.1126/scitranslmed.aaw2607. PubMed PMID: 31316007; PMCID: PMC6788755. PMC6788755 31316007

[207] Hansen SG, Womack J, Scholz I, Renner A, Edgel KA, Xu G, Ford JC, Grey M, St Laurent B, Turner JM, Planer S, Legasse AW, Richie TL, Aguiar JC, Axthelm MK, Villasante ED, Weiss W, Edlefsen PT, Picker LJ, Fruh K. Cytomegalovirus vectors expressing Plasmodium knowlesi antigens induce immune responses that delay parasitemia upon sporozoite challenge. PLoS One. 2019;14(1):e0210252. Epub 2019/01/24. doi: 10.1371/journal.pone.0210252. PubMed PMID: 30673723; PMCID: PMC6343944. 30673723 PMC6343944

[208] Murray SE, Nesterenko PA, Vanarsdall AL, Munks MW, Smart SM, Veziroglu EM, Sagario LC, Lee R, Claas FHJ, Doxiadis IIN, McVoy MA, Adler SP, Hill AB. Fibro-blast-adapted human CMV vaccines elicit predominantly conventional CD8 T cell responses in humans. The Journal of experimental medicine. 2017;214(7):1889-99. Epub 2017/06/02. doi: 10.1084/jem.20161988. PubMed PMID: 28566275; PMCID: PMC5502433. 28566275 PMC5502433

[209] Marshall EE, Malouli D, Hansen SG, Gilbride RM, Hughes CM, Ventura AB, Ainslie E, Selseth AN, Ford JC, Burke D, Kreklywich CN, Womack J, Legasse AW, Axthelm MK, Kahl C, Streblow D, Edlefsen PT, Picker LJ, Fruh K. Enhancing safety of cytomegalovirus-based vaccine vectors by engaging host intrinsic immunity. Science translational medicine. 2019;11(501). Epub 2019/07/19. doi: 10.1126/scitranslmed.aaw2603. PubMed PMID: 31316006; PMCID: PMC6830438. PMC6830438 31316006

[210] Hansen SG, Sacha JB, Hughes CM, Ford JC, Burwitz BJ, Scholz I, Gilbride RM, Lewis MS, Gilliam AN, Ventura AB, Malouli D, Xu G, Richards R, Whizin N, Reed JS, Hammond KB, Fischer M, Turner JM, Legasse AW, Axthelm MK, Edlefsen PT, Nelson JA, Lifson JD, Fruh K, Picker LJ. Cytomegalovirus vectors violate CD8+ T cell epitope recognition paradigms. Science. 2013;340(6135):1237874. Epub 2013/05/25. doi: 10.1126/science.1237874. PubMed PMID: 23704576; PMCID: PMC3816976. 23704576 PMC3816976

[211] Hansen SG, Powers CJ, Richards R, Ventura AB, Ford JC, Siess D, Axthelm MK, Nelson JA, Jarvis MA, Picker LJ, Fruh K. Evasion of CD8+ T cells is critical for superinfection by cytomegalovirus. Science. 2010;328(5974):102-6. Epub 2010/04/03. doi: 10.1126/science.1185350. PubMed PMID: 20360110; PMCID: PMC2883175. 20360110 PMC2883175

[212] Hansen SG, Wu HL, Burwitz BJ, Hughes CM, Hammond KB, Ventura AB, Reed JS, Gilbride RM, Ainslie E, Morrow DW, Ford JC, Selseth AN, Pathak R, Malouli D, Legasse AW, Axthelm MK, Nelson JA, Gillespie GM, Walters LC, Brackenridge S, Sharpe HR, Lopez CA, Fruh K, Korber BT, McMichael AJ, Gnanakaran S, Sacha JB, Picker LJ. Broadly targeted CD8(+) T cell responses restricted by major histo-compatibility complex E. Science. 2016;351(6274):714-20. Epub 2016/01/23. doi: 10.1126/science.aac9475. PubMed PMID: 26797147; PMCID: PMC4769032. 26797147 PMC4769032

[213] Furman D, Jojic V, Sharma S, Shen-Orr SS, Angel CJ, Onengut-Gumuscu S, Kidd BA, Maecker HT, Concannon P, Dekker CL, Thomas PG, Davis MM. Cytomegalovirus infection enhances the immune response to influenza. Science translational medicine. 2015;7(281):281ra43. Epub 2015/04/04. doi: 10.1126/scitranslmed.aaa2293. PubMed PMID: 25834109; PMCID: PMC4505610. PMC4505610 25834109

[214] Barton ES, White DW, Cathelyn JS, Brett-McClellan KA, Engle M, Diamond MS, Miller VL, Virgin HWt. Herpesvirus latency confers symbiotic protection from bacterial infection. Nature. 2007;447(7142):326-9. Epub 2007/05/18. doi: 10.1038/nature05762. PubMed PMID: 17507983. 17507983

